# Exposure to select PFAS and PFAS mixtures alters response to platinum-based chemotherapy in endometrial cancer cell lines

**DOI:** 10.1186/s12940-023-01034-2

**Published:** 2023-12-14

**Authors:** Brittany P. Rickard, Marta Overchuk, Justin Tulino, Xianming Tan, Frances S. Ligler, Victoria L. Bae-Jump, Suzanne E. Fenton, Imran Rizvi

**Affiliations:** 1 Curriculum in Toxicology & Environmental Medicine, University of North Carolina School of Medicine, University of North Carolina at Chapel Hill, 116 Manning Drive, Chapel Hill, NC 27599 USA; 2 Mechanistic Toxicology Branch, Division of Translational Toxicology, National Institute of Environmental Health Sciences, 111 T.W. Alexander Drive, Research Triangle Park, Durham, NC 27709 USA; 3 Joint Department of Biomedical Engineering, University of North Carolina at Chapel Hill, 116 Manning Drive, Chapel Hill, NC 27599, USA; Engineering Building III, North Carolina State University, Raleigh, NC 27606 USA; 4 Department of Biostatistics, University of North Carolina School of Public Health, 135 Dauer Drive, Chapel Hill, NC 27599 USA; 5 Department of Biomedical Engineering, Texas A&M University, 101 Bizzell Street, College Station, TX 77843 USA; 6 Lineberger Comprehensive Cancer Center, University of North Carolina School of Medicine, 450 West Drive, Chapel Hill, NC 27599 USA; 7 Division of Gynecologic Oncology, University of North Carolina at Chapel Hill, 101 Manning Drive, Chapel Hill, NC 27599 USA; 8 Center for Environmental Health and Susceptibility, University of North Carolina at Chapel Hill, 135 Dauer Drive, Chapel Hill, NC 27599 USA

**Keywords:** PFAS, Endometrial cancer, Therapy resistance, Carboplatin, Cisplatin, Mitochondria

## Abstract

**Background:**

Exposure to per- and poly-fluoroalkyl substances (PFAS) has been associated with significant alterations in female reproductive health. These include changes in menstrual cyclicity, timing of menarche and menopause, and fertility outcomes, as well as increased risk of endometriosis, all of which may contribute to an increased risk of endometrial cancer. The effect of PFAS on endometrial cancer cells, specifically altered treatment response and biology, however, remains poorly studied. Like other gynecologic malignancies, a key contributor to lethality in endometrial cancer is resistance to chemotherapeutics, specifically to platinum-based agents that are used as the standard of care for patients with advanced-stage and/or recurrent disease.

**Objectives:**

To explore the effect of environmental exposures, specifically PFAS, on platinum-based chemotherapy response and mitochondrial function in endometrial cancer.

**Methods:**

HEC-1 and Ishikawa endometrial cancer cells were exposed to sub-cytotoxic nanomolar and micromolar concentrations of PFAS/PFAS mixtures and were treated with platinum-based chemotherapy. Survival fraction was measured 48-h post-chemotherapy treatment. Mitochondrial membrane potential was evaluated in both cell lines following exposure to PFAS ± chemotherapy treatment.

**Results:**

HEC-1 and Ishikawa cells displayed differing outcomes after PFAS exposure and chemotherapy treatment. Cells exposed to PFAS appeared to be less sensitive to carboplatin, with instances of increased survival fraction, indicative of platinum resistance, observed in HEC-1 cells. In Ishikawa cells treated with cisplatin, PFAS mixture exposure significantly decreased survival fraction. In both cell lines, increases in mitochondrial membrane potential were observed post-PFAS exposure ± chemotherapy treatment.

**Discussion:**

Exposure of endometrial cancer cell lines to PFAS/PFAS mixtures had varying effects on response to platinum-based chemotherapies. Increased survival fraction post-PFAS + carboplatin treatment suggests platinum resistance, while decreased survival fraction post-PFAS mixture + cisplatin exposure suggests enhanced therapeutic efficacy. Regardless of chemotherapy sensitivity status, mitochondrial membrane potential findings suggest that PFAS exposure may affect endometrial cancer cell mitochondrial functioning and should be explored further.

**Supplementary Information:**

The online version contains supplementary material available at 10.1186/s12940-023-01034-2.

## Introduction

Environmental exposure to per- and poly-fluoroalkyl substances (PFAS) contributes to the onset and progression of various disease states, including those of reproductive system [1, 2]. In women, many adverse reproductive effects have been associated with PFAS exposure including disrupted menstrual cyclicity [3, 4], preeclampsia [5, 6], and shorter lactation duration[7, 8]. Several studies have also shown that higher PFAS exposure levels are associated with endometriosis [9, 10], which can be a precursor to endometrial cancer [11, 12]. A study by Campbell et al*.* [9] found that blood levels of perfluorooctanoic acid (PFOA), perfluorooctane sulfonate (PFOS), and perfluorononanoic acid (PFNA) were elevated in women reporting endometriosis. In another study evaluating the association between PFAS and endometriosis in women from the Salt Lake City and San Francisco Bay areas, PFOA and PFNA were also reported to be associated with endometriosis, while serum PFOS and PFOA levels increased the odds for more severe disease [10]. PFAS exposure, specifically perfluorobutane sulfonic acid (PFBS), has also been linked to endometriosis-related infertility in a study by Wang et al*. *[13]. These findings are critical, as PFAS exposure has recently been linked with increased adiposity [14, 15], the main risk factor for endometrial cancer, due to the excess conversion of androgens to estrone in adipose tissue [16]. Despite strong evidence that PFAS target the female reproductive system [2], little is known about the risk of gynecologic cancers following chronic PFAS exposure. 

For reasons that are not yet understood, endometrial cancer is on the rise in the United States and is one of the few cancers that had a positive annual percent change in death rate from 2015—2019 (1.9% per year) [17–19]. It is also the most common gynecologic cancer with an estimated 66,200 new cases expected in 2023 [17, 20]. Endometrial adenocarcinomas, which are predominantly endometrioid in histology, are referred to as type 1 endometrial cancers and represent ~ 90% of cases, while non-endometrial type 2 disease comprises serous or clear cell histology tumors [21, 22]. These cancers are largely diagnosed at an early stage, with as many as 84% of patients being diagnosed at stages I-II [18, 21]. Surgical resection of the uterus, fallopian tubes, and ovaries (total hysterectomy bilateral salpingo-oophorectomy) is, in most cases, considered the first line of therapy against endometrial cancer [23, 24]. In cases of early reproductive age endometrial cancer, which is also on the rise, medroxyprogesterone acetate and/or levonorgestrel intrauterine system is favored to preserve fertility [25]. Fertility preservation is also favored in the treatment of endometrial intraepithelial neoplasia, which although precancerous, may develop into endometrial cancer later in life.

The role of chemotherapy in endometrial cancer is more prominent in patients with advanced-stage, metastatic, and/or recurrent disease [21, 24]. Like other gynecologic malignancies, multiagent chemotherapy used for endometrial cancer involves platinum- and taxane-based agents, in addition to doxorubicin [21, 24]. However, low initial response rates to multiagent chemotherapy remain an issue and are suggestive of platinum resistance [21], which is responsible for 90% of treatment failures in endometrial cancer [26–28].Thus, understanding factors responsible for the development of and identifying methods to overcome chemotherapy resistance are critical for improving patient outcomes.

In the context of cancer, alterations in mitochondrial death pathways are known to underlie chemoresistance [29]. It is also known that compared to healthy cells, cancer cells have an increased number of mutations in mitochondrial deoxyribose nucleic acid (mtDNA), leading to mitochondrial dysfunction [30]. These mtDNA mutations can often promote mitochondrial bioenergetic reprogramming, a hallmark of cancer [31], and upregulation of cancer-associated signaling pathways. In our recently published study [32], select PFAS were found to be associated with carboplatin resistance in ovarian cancer cells, for which the standard of care is also combination therapy using platinum- and taxane-based agents [33, 34]. To our knowledge, this is the only study to date reporting an association between PFAS exposure and resistance to chemotherapy. To further address this critical knowledge gap, this study sought to evaluate the contribution of PFAS exposure to therapy resistance in the context of other gynecologic malignancies. The goal of this study was to evaluate how acute exposure to PFAS, legacy and emerging compounds, and PFAS mixtures relevant to North Carolina water supplies [35], and most other U.S. states [36], affect endometrial cancer response to platinum-based chemotherapies and mitochondrial function.

In the present study, HEC-1B (referred to as HEC-1) and Ishikawa cells were exposed to PFOA, perfluoroheptanoic acid (PFHpA), perfluoropentanoic acid (PFPA), or mixtures thereof in concentrations ranging from 0.025 – 2.25 μM. Nanomolar and micromolar concentrations of PFAS were selected to maintain human relevance; however, it is important to note that in the context of endometrial cancer, reference doses for PFOA, PFHpA, PFPA, and PFAS mixtures have not yet been established. In highly contaminated communities (North Carolina, USA; West Virginia, USA; Venice, Italy), serum concentrations of PFOA, PFHpA, and PFPA range from 0.0001 μg/mL to 17.6 μg/mL [37–39]. The concentrations of 0.025 – 2 μM PFOA, PFHpA, and PFPA used in the present study can be translated to 0.010—0.828 μg/mL, 0.009—0.728 μg/mL, and 0.007—0.528 μg/mL [32], respectively, highlighting the overlap with those reported in epidemiologic studies.

HEC-1 and Ishikawa cell lines are both representative of endometrial adenocarcinomas with endometrioid histology, the most common histologic subtype of endometrial cancer. Survival fraction, defined as the fraction of viable cells normalized to the vehicle control, was measured post-PFAS exposure, and sub-cytotoxic concentrations were selected for subsequent experiments. Endometrial cancer cells exposed to PFAS were then treated with platinum-based chemotherapy, either carboplatin or cisplatin, over a range of doses. Based on prior experience [32], the central hypothesis of this study is that select PFAS induce resistance to platinum-based chemotherapy in endometrial cancer cells. The relationship between PFAS exposure and mitochondrial membrane potential (ΔΨ_m_) pre- and post-PFAS exposure ± carboplatin or cisplatin was also examined, since altered mitochondrial function has been associated with therapy resistance in cancer [29, 30, 32, 40].

## Methods

### Cell culture

Human endometrioid endometrial adenocarcinoma HEC-1 and Ishikawa cell lines were obtained from the lab of Dr. Victoria Bae-Jump and the European Collection of Authenticated Cell Cultures (ECACC), respectively. HEC-1 cells were grown in McCoy’s 5A medium (American Type Culture Collection, Manassas, VA, USA) supplemented with 10% fetal bovine serum (FBS, Cytiva HyClone™, Marlborough, MA, USA), 2 mM L-glutamine (Corning, Corning, NY, USA), and 1% antibiotic–antimycotic solution (Corning). Ishikawa cells were grown in Minimum Essential Medium with Earle’s Balanced Salt Solution (MEM/EBSS, Cytiva HyClone™) supplemented with 5% FBS, 1% non-essential amino acids (Gibco, Billings, MT, USA), 2 mM L-glutamine, 100 U/mL penicillin and 100 μg/mL streptomycin (Sigma-Aldrich, St. Louis, MO, USA). Cells were maintained in monolayers at 37 °C in a humidified incubator with 5% CO_2_ and routinely tested for mycoplasma contamination using the MycoAlert™ PLUS Kit (Lonza Bioscience, Basel, Switzerland, Catalog #LT07-710). Cells were discarded at passage 30 and new stocks were thawed. The HEC-1B cell line was authenticated by the Virology Core at the University of North Carolina at Chapel Hill using Ion Torrent Precision ID GlobalFilerTM Next Generation Sequencing Short Team Repeat Panel (Applied Biosystems, Waltham, MA, USA). The Ishikawa cell line was authenticated by ECACC prior to arrival.

### Preparation of PFAS stocks

PFAS stock solutions were prepared as described previously [32, 41]. Briefly, PFOA (CAS#335–67-1) was obtained from Synquest Laboratories (Alachua, FL, USA, Catalog #2121–3-18, 98% purity) in powder form, PFHpA (CAS#375–85-9) was obtained from Sigma-Aldrich (Catalog#342,041-5G, 97% purity) in powder form, and PFPA was obtained from TCI America (Portland, OR, USA, Catalog #N06055G, 98% purity) in liquid form. 10 mM stocks of each chemical were prepared in potassium hydroxide in methanol (Lab Chem Inc., Zelienople, PA, USA, Catalog #LC195402). Solution without PFAS is referred to as “methanol” or “vehicle control”. All PFAS stock solutions were stored at -20 °C. Media supplemented with an additional 25 mM N-2-hydroxyethylpiperazine-N'-2-ethanesulfonic acid (HEPES) was used to maintain the pH when basic methanol was added.

### Evaluation of methanol cytotoxicity

To determine the optimal concentration of methanol for PFAS dosing while minimizing toxicity, HEC-1 and Ishikawa cells were seeded at a density of 2,500 cells/well. Corning 96-well white-walled plates were used for all experiments measuring survival fraction. These seeding densities were selected based on experiments exploring the linear dynamic range of the CellTiter Glo Luminescent Cell Viability Assay (Promega Corp., Madison, WI, USA) for each cell line after 6 days (Figure S1). Once the optimal cell densities were determined, cells were plated for methanol dose-ranging experiments. As previously described [32], cells were treated with concentrations of 0–5% methanol in serum-free media 24-h post-plating. Serum-free medium for HEC-1 cells was McCoy’s 5A medium supplemented with 2 mM L-glutamine and 1% antibiotic–antimycotic solution. Serum-free medium for Ishikawa cells was MEM/EBSS supplemented with 1% non-essential amino acids, 2 mM L-glutamine, 100 U/mL penicillin and 100 μg/mL streptomycin. Since some PFAS, including chemicals tested in this study, were reported to bind serum [42], a 1-h serum-free pulse was used across all experiments to ensure cellular PFAS uptake. After 1-h of methanol exposure in serum-free medium, cells were exposed to methanol in 2X serum-containing medium (complete medium conditions with FBS percentage doubled) for 47 h, for a total exposure time of 48 h. After 48 h, methanol-containing medium was replaced with fresh medium (no methanol) for 48 h prior to assessing viability using the CellTiter Glo assay. For this assay, 50 μL of medium was removed from all wells and 50 μL of reconstituted CellTiter Glo reagent was added. Plates were shaken orbitally for 2 min prior to incubation at room temperature for 10 min to let the signal stabilize. After 10 min, the CellTiter Glo luminescent signal was measured using the SpectraMax iD3 plate reader (Molecular Devices, LLC. San Jose, CA, USA). Survival fraction was quantified by normalizing CellTiter Glo luminescent signals from exposure groups to that of controls. At methanol concentrations of 2% and higher, significant decreases (> 10%) in survival fraction were observed in both cell lines; however, up to 1% methanol was tolerated (Figure S2). Thus, 1% methanol was selected as the optimal concentration for the remainder of experiments.

### Evaluation of PFAS and PFAS mixture cytotoxicity

HEC-1 and Ishikawa cells were seeded at 2,500 cells/well in Corning 96-well white-walled plates for 24 h prior to exposure to PFAS. As described previously [32], on the day of exposure, PFAS solutions were prepared from 10 mM stock solutions at concentrations ranging from 25 – 1000 μM. Final dosing solutions were prepared at concentrations ranging from 0.025 – 2 μM (1% methanol) in serum-free medium for each cell line. PFAS were dosed in serum-free medium for 1 h, followed by exposure in 2X serum-containing medium for 47 h. After PFAS exposure for 48 h, PFAS-containing medium was removed and replaced with fresh medium (no PFAS, complete medium) for 48 h prior to reading cell viability using the CellTiter Glo assay. To evaluate the cytotoxicity (defined as > 10% decrease in survival fraction) of PFAS mixtures, stock solutions were prepared at concentrations ranging from 10 – 1000 μM. Final dosing solutions were prepared at concentrations ranging from 0.3—2.25 μM total PFAS, depending on the number of agents used in the solution. For example, 2 μM mixtures (PFOA + PFHpA, PFOA + PFPA, and PFHpA + PFPA) were made using 2 individual PFAS chemicals at a concentration of 1 μM each while 2.25 μM mixtures (PFOA + PFHpA + PFPA) were made using 3 individual PFAS chemicals at concentrations of 0.75 μM each. Timelines of exposure and cell viability measurement methods for PFAS mixtures are the same as those described for PFAS exposure.

### Evaluation of chemotherapy response pre- and post-PFAS exposure

In this study, both the efficacies of carboplatin and cisplatin were evaluated in HEC-1 and Ishikawa cell lines. HEC-1 and Ishikawa cells were plated at 2,500 cells/well in 96-well plates and allowed to adhere for 24 h prior to PFAS exposure. PFAS solutions were prepared and dosed as described in the previous section. Based on cytotoxicity experiments, only concentrations of 0.5 μM and 2 μM were tested for individual PFAS chemicals, while mixtures were tested at 2 μM (2 PFAS mixture, 1 μM + 1 μM) and 2.25 μM (3 PFAS mixture, 0.75 μM + 0.75 μM + 0.75 μM). After a 1-h serum-free pulse, PFAS were exposed in 2X serum-containing medium for 47 h. After a total of 48 h of PFAS or PFAS mixture exposure, fresh media (no PFAS) containing a range of 50 – 800 μM carboplatin or 2.5 – 50 μM cisplatin was added to plates. Carboplatin (TCI America) treatment solutions were prepared as described in previous publications [32, 41]. Briefly, a 5 mM solution in media was used to prepare solutions ranging from 50 μM – 400 μM for HEC-1 cells or 100 μM – 800 μM for Ishikawa cells. A cisplatin (Enzo Biochem, Inc. Farmingdale, NY, USA) working solution of 1.665 mM in saline was used to create working solutions of 2.5 μM – 25 μM cisplatin for HEC-1 cells and 5 μM – 50 μM for Ishikawa cells. Cells were treated with carboplatin or cisplatin for 48 h prior to cell viability assessments using the CellTiter Glo assay.

For carboplatin and cisplatin dose–response experiments, which evaluated more concentrations of chemotherapy exposure than those used as controls above, timelines remained the same, but PFAS exposure was not included. For consistency with other timelines, cells were exposed to 1% methanol for 48 h (1 h serum-free pulse + 47 h in 2X serum-containing medium) prior to treatment with chemotherapy. Carboplatin working solutions of 1 μM – 400 μM were prepared in HEC-1 medium or 1 μM – 800 μM in Ishikawa medium using the 5 mM stock solution. Cisplatin working solutions of 0.1 μM – 50 μM were prepared in HEC-1 or Ishikawa medium using the 1.665 mM stock solution. Chemotherapy was administered on the plate for 48 h prior to assessing cell viability using the CellTiter Glo assay. The goal of these dose–response experiments was to determine IC_50_ and IC_90_ values for both chemotherapeutics in HEC-1 and Ishikawa cells.

### Evaluation of endometrial cancer cell ΔΨ_m_ post-PFAS exposure and/or chemotherapy treatment

To measure the effects of PFAS and PFAS mixtures on ΔΨ_m_, HEC-1 and Ishikawa cells were seeded at 20,000 cells/well. Based on previous studies demonstrating that ovarian cancer cells were seeded at 40,000 cells/well [32, 41], 20,000 cells/well seemed sufficient for endometrial cancer cells considering they appeared to grow about twice as fast and were less reliant on cell–cell contact. Cells were allowed to grow for 24 h post-seeding prior to administration of 10 μg/mL 5,5’,6,6’-tetrachloro-1,1′3,3’-tetraethylbenzimidazolocarbo-cyanine iodide (JC-1) dye (Invitrogen, Waltham, MA, USA) for 15 min. After 15 min, JC-1 dye was removed and cells were washed with phosphate-buffered saline (PBS) prior to incubation with PFAS (1 μM or 4 μM PFOA, PFHpA, or PFPA), PFAS mixtures (2 PFAS mixture, 2 μM + 2 μM; 3 PFAS mixture, 1.5 μM + 1.5 μM + 1.5 μM), or 100 μM carbonyl cyanide m-chlorophenyl hydrazone (CCCP, Sigma-Aldrich) in a total of 50 μL serum-free (for PFAS and PFAS mixtures) medium for one hour. Concurrently, medium or chemotherapy (carboplatin or cisplatin) was added to the wells containing PFAS/PFAS mixtures to achieve final PFAS concentrations of 0.5 μM and 2 μM for individual agents, 1 μM + 1 μM for 2 PFAS mixtures, or 0.75 μM + 0.75 μM + 0.75 μM for 3 PFAS mixtures. This one-hour serum-free exposure to both PFAS and chemotherapy combined was performed for consistency with cell viability studies and to ensure adequate cell exposure to PFAS prior to the JC-1 readout. Chemotherapy was not co-incubated with CCCP-treated cells. Medium was added to 0 μM chemotherapy controls. Carboplatin solutions were prepared at concentrations ranging from 100 μM – 800 μM for HEC-1 cells and 200 μM – 1600 μM for Ishikawa cells. When 50 μL was added to cells already containing PFAS, carboplatin concentrations equilibrated to 50 μM – 400 μM for HEC-1 cells and 100 μM – 800 μM for Ishikawa cells, consistent with the above methods. Similarly, cisplatin dosing solutions ranging from 5 μM  – 50 μM were prepared for HEC-1 cells and 10 μM – 100 μM for Ishikawa cells. When added to cells already containing PFAS, cisplatin concentrations equilibrated to 2.5 μM – 25 μM for HEC-1 cells and 5 μM – 50 μM for Ishikawa cells. The effects of chemotherapy alone were also evaluated, and for these experiments, HEC-1 and Ishikawa cells were exposed to 50 μM – 400 μM carboplatin and 2.5 μM – 25 μM cisplatin or 100 μM – 800 μM carboplatin and 5 μM – 50 μM cisplatin, respectively. After 1 h of exposure to PFAS, chemotherapy, CCCP, or simultaneous exposure to PFAS + chemotherapy, the JC-1 red:green aggregate ratio was read using the SpectraMax iD3 fluorescence plate reader (green aggregate—excitation: 488 nm, emission: 529 nm; red aggregate—excitation: 488 nm, emission: 590 nm). All dosing for these experiments was performed in the dark to prevent photobleaching of the JC-1 dye.

### Statistical analysis

**Fig. 1:**
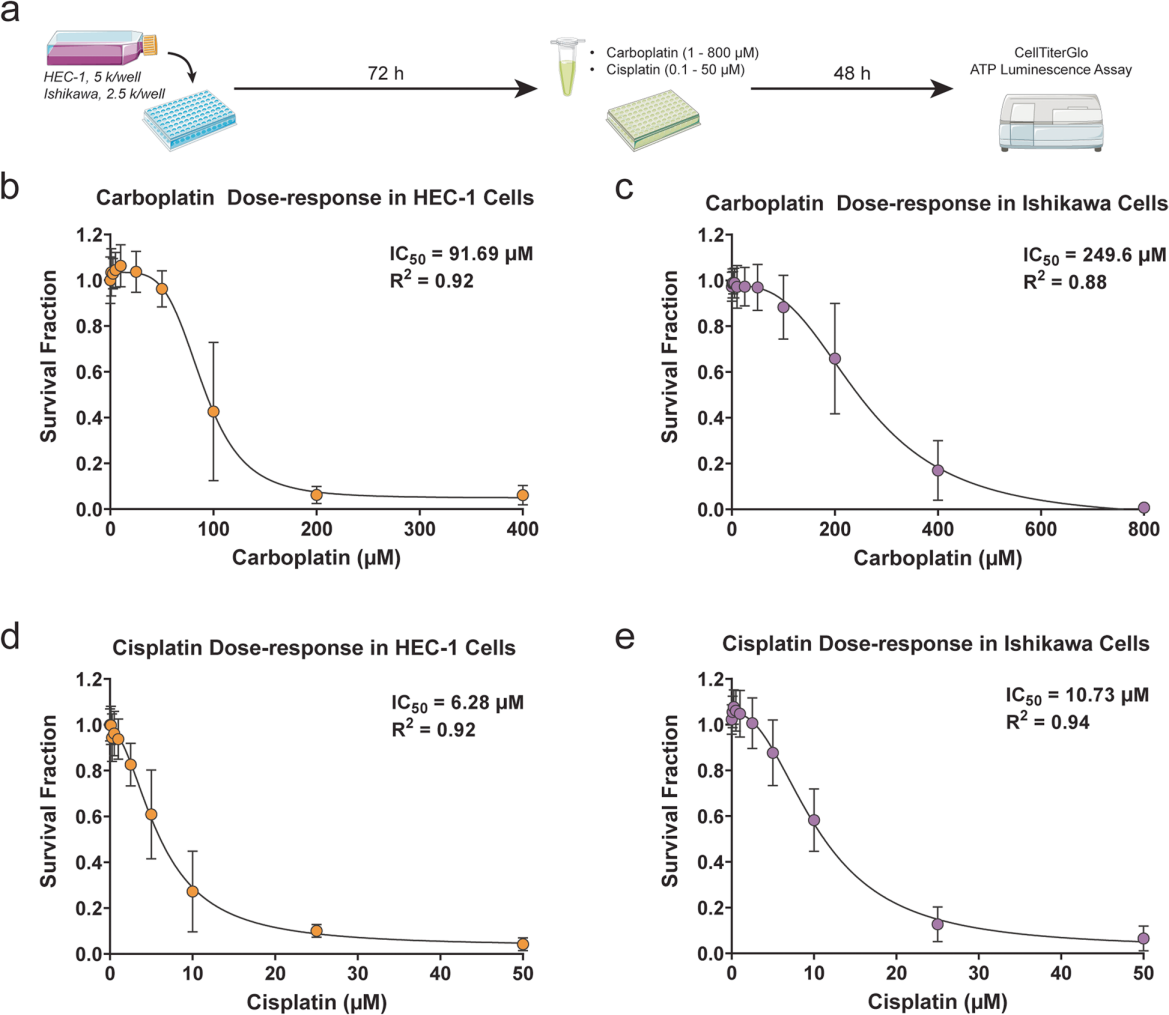
Chemotherapy dose–response in HEC-1 and Ishikawa cells. **a** Timeline of experiments. Dose-dependent reduction in survival fraction in (**b**) HEC-1 (orange) and (**c**) Ishikawa (purple) cells after exposure to 0 – 400 μM or 0 – 800 μM carboplatin for 48 h, respectively. Dose-dependent reduction in survival fraction in (**d**) HEC-1 and (**e**) Ishikawa cells after exposure to 0.1 – 50 μM cisplatin for 48 h. These results indicate chemotherapy sensitivity in both cell lines at baseline. *n* = 4 independent experiments in triplicate for HEC-1 carboplatin curves, *n* = 4 independent experiments in sextuplicate for Ishikawa carboplatin curves (*n* = 2 in sextuplicate for 1 μM and 800 μM groups), *n* = 3 independent experiments in sextuplicate for HEC-1 cisplatin curves, and *n* = 4 independent experiments in sextuplicate for Ishikawa cisplatin curves

**Fig. 2:**
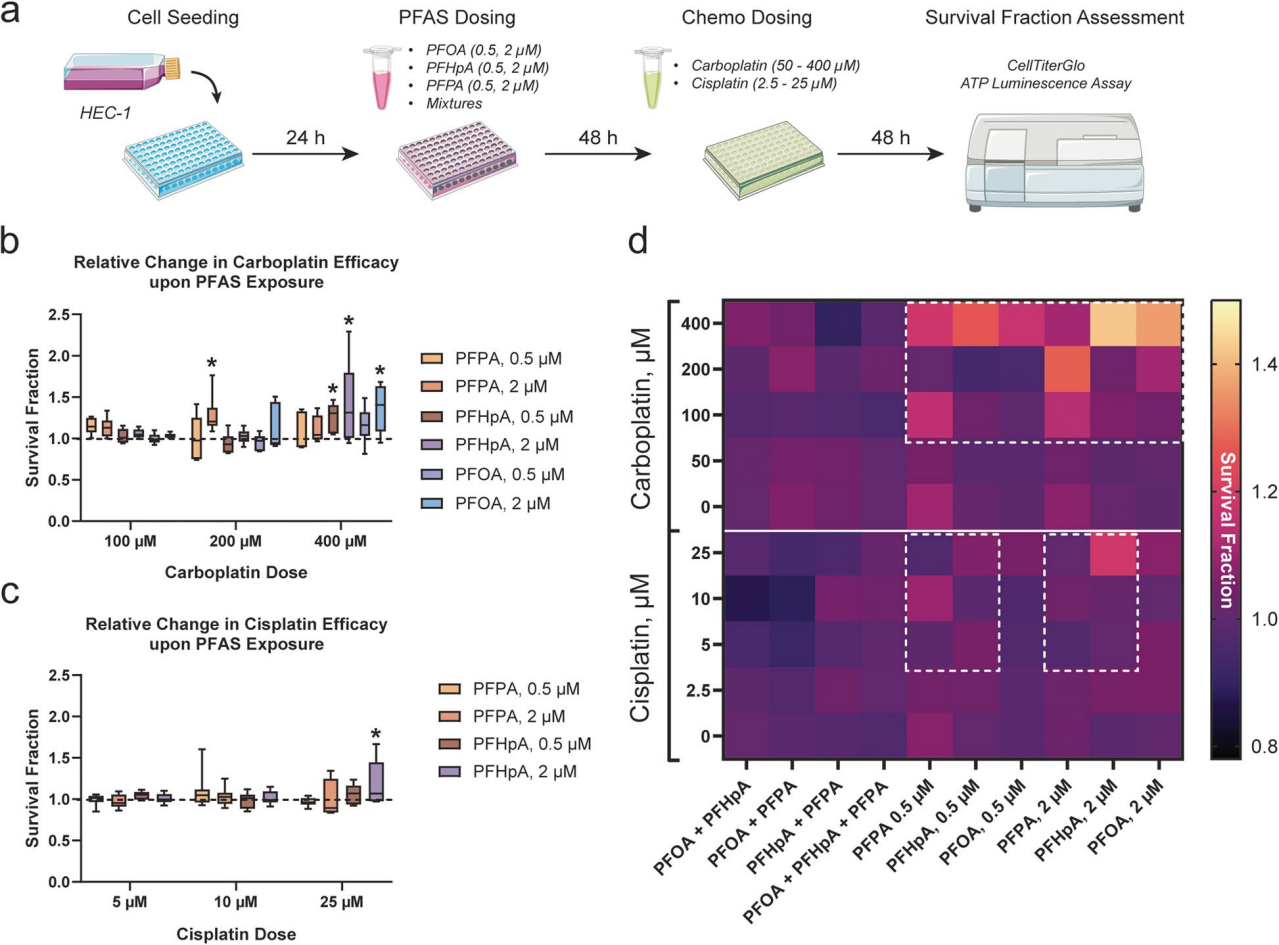
Exposure to PFAS impacts survival fraction in HEC-1 cells. **a** Timeline of experiments. **b** Survival fraction significantly increased compared to controls (dashed line) in HEC-1 cells exposed to select PFAS then treated with carboplatin. **c** A significant increase in survival fraction was observed in HEC-1 cells exposed to PFHpA then treated with cisplatin. **d** Heat map of changes in survival fraction after exposure to PFAS or PFAS mixtures followed by treatment with platinum-based chemotherapy. White-dashed boxes in the top panel (carboplatin-treated) highlight groups in which significant increases in survival fraction were observed, representing chemotherapy resistant cell populations. In the bottom panel (cisplatin-treated), a significant change was only observed in one group (lightest pink box). Data in graphs (b) and (c) are shown as a percentage of the vehicle control for each chemotherapy group; *n* = 3 independent experiments in duplicate. Significant differences between PFAS or PFAS mixture exposure groups versus vehicle group are denoted by * (*p* < 0.05). The entire dataset is included in Supplemental Figs. 5 and 9

**Fig. 3:**
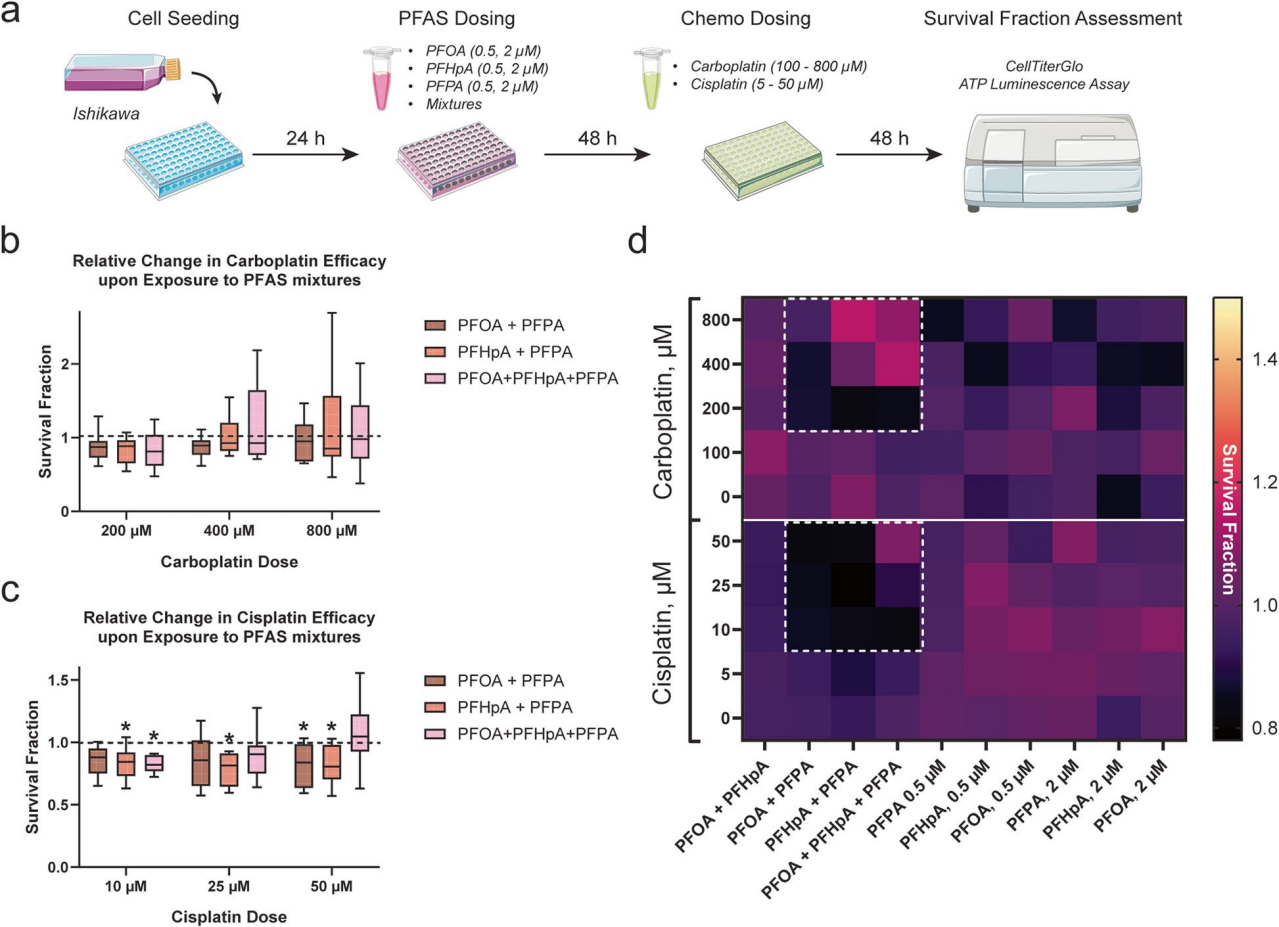
Exposure to PFAS mixtures impacts survival fraction in Ishikawa cells. **a** Timeline of experiments. **b** Survival fraction is not significantly altered compared to controls (dashed line) in Ishikawa cells exposed to PFAS mixtures and treated with carboplatin. **c** Relative to the vehicle control, a significant reduction in survival fraction was observed in Ishikawa cells exposed to select PFAS mixtures then treated with cisplatin, indicative of an enhanced chemotherapeutic response. **d** Heat map of changes in survival fraction after exposure to PFAS or PFAS mixtures followed by treatment with platinum-based chemotherapy. Darker black boxes in the bottom panel (cisplatin-treated) highlight groups in which significant decreases in survival fraction were observed, in contrast to those in the top panel (carboplatin-treated) where no significant changes were observed. Data in graphs (b) and (c) are shown as a percentage of the vehicle control for each chemotherapy group; *n* = 4 independent experiments in duplicate. Significant differences between PFAS or PFAS mixture exposure group versus vehicle group are denoted by * (*p* < 0.05). The entire dataset is provided in Supplemental Figs. 6 and 10

**Fig. 4:**
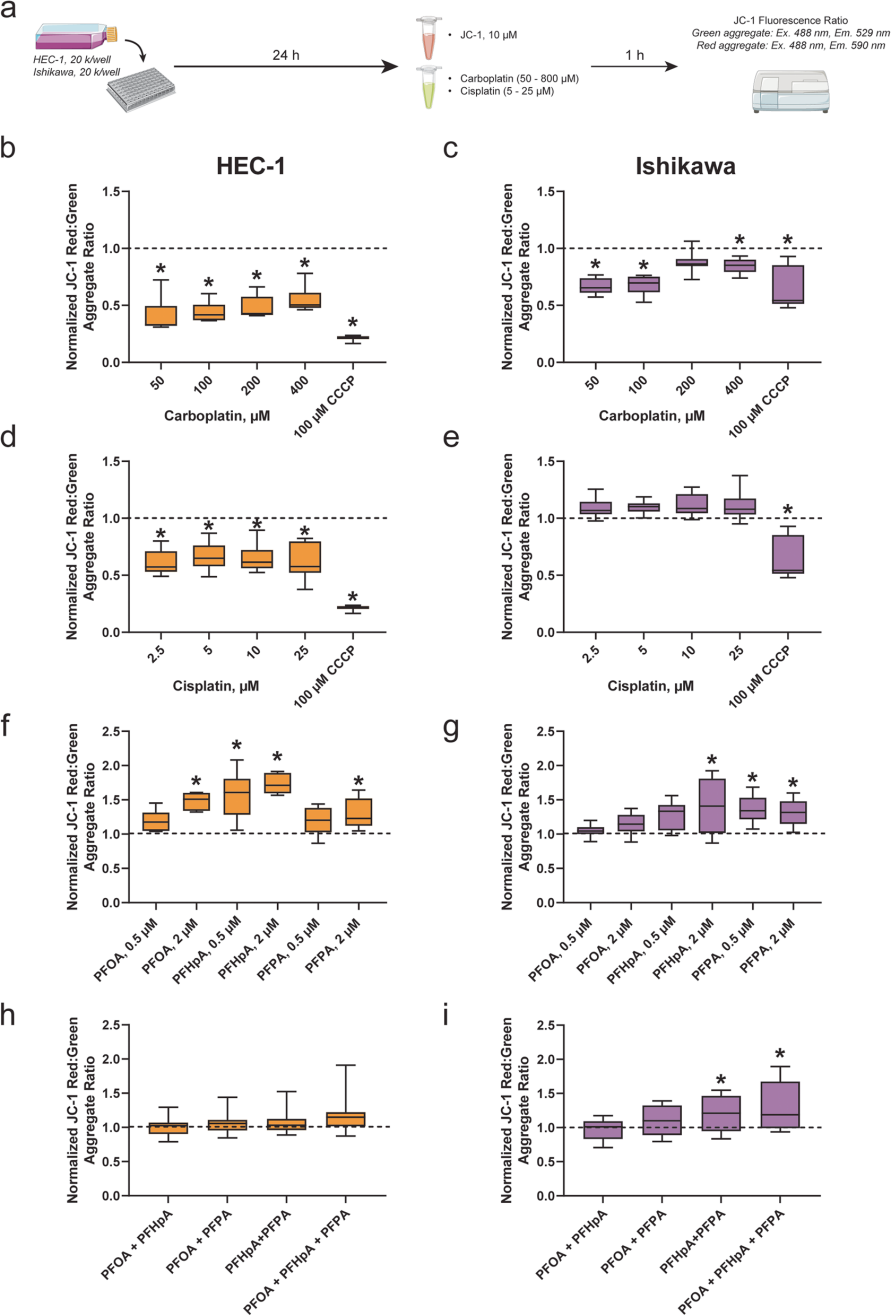
ΔΨ_m_ increases after exposure to PFAS in HEC-1 and Ishikawa cells but decreases after treatment with platinum-based chemotherapy. **a** Timeline of experiments. After treatment with carboplatin, cisplatin, and/or CCCP, ΔΨ_m_ decreased significantly compared to the vehicle control in (**b**,**d**) HEC-1 and (**c**,**e**) Ishikawa cells. In (**f**) HEC-1 (orange) and (**g**) Ishikawa (purple) cells exposed to select PFAS, ΔΨ_m_ increases significantly compared to the vehicle control (dashed line). Exposure to PFAS mixtures do not significantly alter ΔΨ_m_ in (**h**) HEC-1 cells. In (**i**) Ishikawa cells, a significant increase ΔΨ_m_ was observed in select groups. Data are shown as a percentage of the vehicle control; *n* = 3 in at least triplicate for HEC-1 and Ishikawa carboplatin (**b**,**c**) and cisplatin (**d**,**e**), *n* = 3 independent experiments in duplicate for (**f**) HEC-1 PFAS, *n* = 4 independent experiments in duplicate for (**g**) Ishikawa PFAS, *n* = 4 independent experiments in at least triplicate for (**h**) HEC-1 mixtures and (**i**) Ishikawa mixtures. Significant differences between PFAS, PFAS mixtures, or chemotherapy/CCCP group versus vehicle group are denoted by * (*p* < 0.05)

**Fig. 5:**
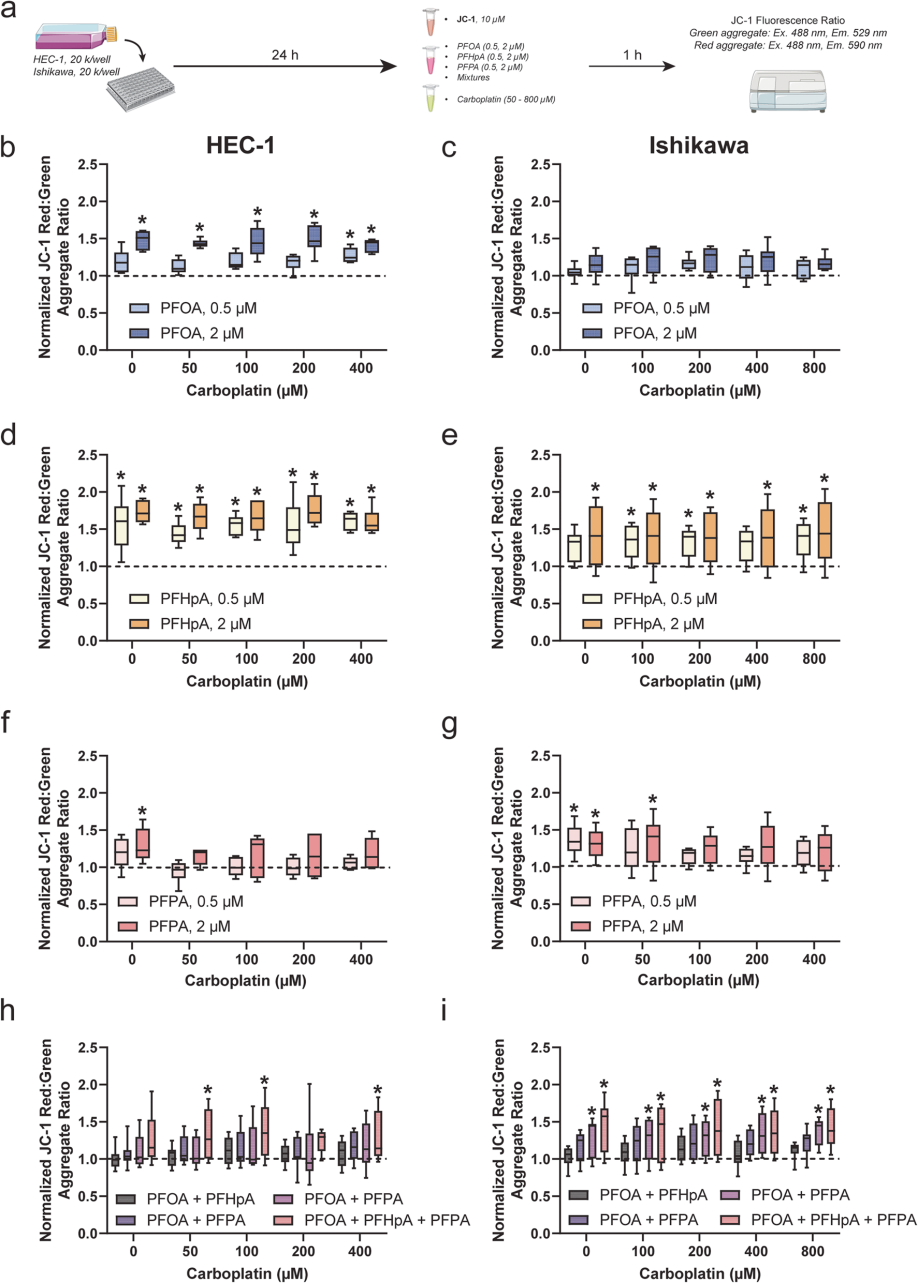
ΔΨ_m_ increases in HEC-1 and Ishikawa cells following exposure to PFAS or PFAS mixtures + carboplatin treatment. **a** Timeline of experiments. In (**b**,**d**,**f**) HEC-1 cells, ΔΨ_m_ increased in cells exposed to select PFAS after treatment with carboplatin. A significant increase in ΔΨ_m_ after exposure of HEC-1 cells to (**h**) PFAS mixtures was only observed in the PFOA + PFHpA + PFPA group. In Ishikawa cells, (**c**) PFOA exposure did not significantly alter ΔΨ_m_. Increased ΔΨ_m_ was observed in Ishikawa cells exposed to (**e**) PFHpA and (**g**) PFPA at numerous carboplatin treatment levels. Increased ΔΨ_m_ was also observed in Ishikawa cells after exposure to (**i**) PFAS mixtures then treatment with carboplatin. Data are shown as a percentage of the vehicle control at each carboplatin level; *n* = 3 independent experiments in duplicate for HEC-1 individual PFAS, *n* = 4 independent experiments in duplicate for HEC-1 mixtures, Ishikawa individual PFAS, and Ishikawa mixtures. Significant differences between PFAS/PFAS mixture + carboplatin treatment group versus vehicle group at each respective carboplatin dose are denoted by * (*p* < 0.05)

**Fig. 6:**
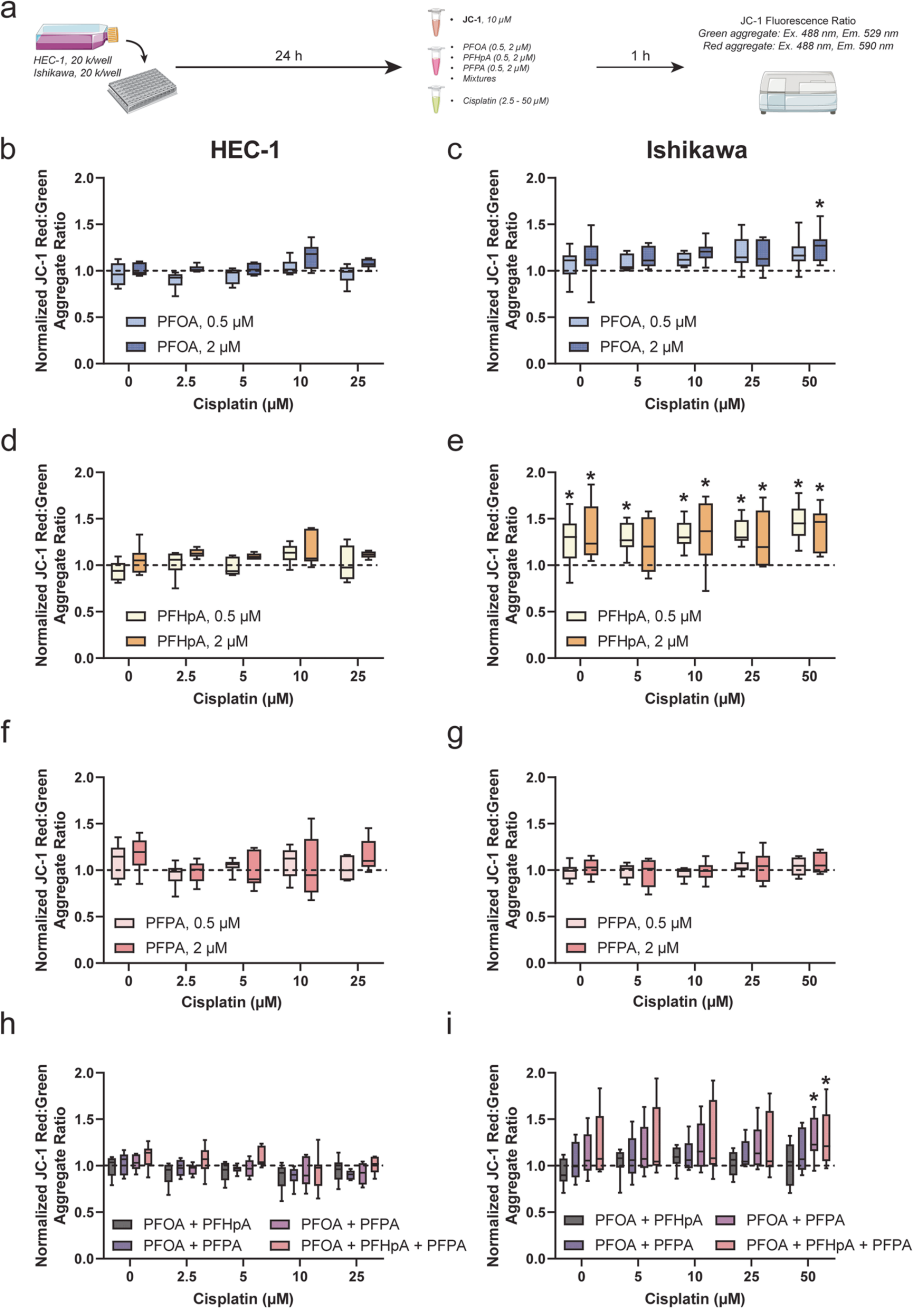
ΔΨ_m_ increases in Ishikawa, but not HEC-1, cells following exposure to PFAS or PFAS mixtures + cisplatin treatment. **a** Timeline of experiments. In HEC-1 cells, ΔΨ_m_ was unchanged compared to the vehicle control in all groups exposed to (**b**,**d**,**f**) PFAS and (**h**) PFAS mixtures then treated with cisplatin. ΔΨ_m_ increased in Ishikawa cells exposed to (**c**) PFOA, (**e**) PFHpA, and (**i**) PFAS mixtures. No changes in ΔΨ_m_ were observed in Ishikawa cells exposed to (**g**) PFPA. Data are shown as a percentage of the vehicle control at each cisplatin level; *n* = 3 or 4 independent experiments in duplicate for HEC-1 cells, *n* = 4 independent experiments for Ishikawa cells. Significant differences between PFAS/PFAS mixture + carboplatin treatment group versus vehicle group at each respective cisplatin dose are denoted by * (*p* < 0.05)

To evaluate the effect of PFAS concentration and chemotherapy concentration on outcomes of interest, such as survival fraction and ΔΨ_m_, unpaired t-tests, one-way or two-way ANOVA, or linear/nonlinear regression were employed, as appropriate and as described in our previous study [32]. To compare outcomes between two groups (e.g., PFAS-exposed cells vs. vehicle control under a given chemotherapy concentration), corresponding contrasts were extracted from linear regression analysis. Linear or nonlinear regression analyses were also performed in chemotherapy dose-responses and cell density linear dynamic range experiments to attain R^2^ values and fitted curves. All tests are 2-sided at alpha level 0.05 unless otherwise specified. All analyses were performed in R 4.1.1 [43] or Prism 9.0 software (GraphPad, San Diego, CA, USA). Specific analyses performed are as follows: non-linear regression and unpaired t-tests—Fig. 1; 2-way ANOVA with Dunnett’s multiple comparisons test—Figs. 2, 3, 5, 6, S5, S6, S9, S10, S11, S12; ordinary one-way ANOVA with Dunnett’s multiple comparisons test—Figs. 4, S3, S4, S7, S8; simple linear regression—Figure S1; and 2-way ANOVA with Šídák's multiple comparisons test—Figure S2. All statistics displayed in the main text and supplement were performed using Prism 9.0, but an alternative analysis using a custom R script along with the raw data is provided in the data sharing CEBS link.

## Results

### HEC-1 and Ishikawa cell sensitivity to platinum-based chemotherapies

Since advanced-stage or recurrent endometrial cancer is commonly treated using platinum-based chemotherapy, the dose-dependent effects of carboplatin or cisplatin on survival fraction were evaluated in monolayer cultures of HEC-1 or Ishikawa cells. For HEC-1 cells, carboplatin doses ranging from 1 μM – 400 μM were tested, while Ishikawa cells required a larger dose range of 1 μM – 800 μM to achieve a complete response (Fig. 1a-c). A significant decrease in survival fraction was observed at 100 μM carboplatin for each cell line, with further reduction at higher doses. Specifically, in HEC-1 cells the carboplatin IC_50_ concentration was 91.7 μM, while that of Ishikawa cells was 249.6 μM. IC_90_ values for HEC-1 and Ishikawa cells were 149.6 μM and 538.6 μM, respectively. In cisplatin-treated cells, survival fraction began decreasing significantly at 2.5 μM for HEC-1 cells and 5 μM for Ishikawa cells (Fig. 1d,e). IC_50_ values for HEC-1 and Ishikawa cells treated with cisplatin were 6.3 μM and 10.7 μM, respectively, while IC_90_ values were 19.3 μM and 28.6 μM, respectively. In both cell lines, carboplatin and cisplatin effectively reduced survival fraction in the absence of PFAS exposure.

### Effectiveness of platinum-based chemotherapies in HEC-1 and Ishikawa cells post-PFAS or PFAS mixture exposure

Resistance to platinum-based chemotherapy is a major contributor to disease lethality in advanced-stage endometrial cancer patients. As a result, evaluating the contribution of environmental exposures to diminished treatment response is critical. HEC-1 or Ishikawa cells were exposed to select PFAS at either 0.5 μM or 2 μM. PFAS concentrations were determined based on dose-ranging toxicity experiments, which showed that in both HEC-1 (Figure S3) and Ishikawa cells (Figure S4), all concentrations tested were sub-cytotoxic. In fact, at select nanomolar and micromolar concentrations of PFOA, PFHpA, or PFPA, survival fraction increased significantly compared to controls, suggesting a proliferative effect. HEC-1 cells were then treated with either 0 – 400 μM carboplatin or 0 – 25 μM cisplatin, whereas Ishikawa cells were treated with either 0 – 800 μM carboplatin or 0 – 50 μM cisplatin.

In HEC-1 cells exposed to PFAS then treated with carboplatin, significant increases in survival fraction were observed compared to controls (Figs. 2, S5). Survival fraction increased significantly compared to the vehicle control in HEC-1 cells exposed 2 μM PFPA + 200 μM carboplatin (1.28 ± 0.24), 0.5 μM PFHpA + 400 μM carboplatin (1.26 ± 0.17), 2 μM PFHpA + 400 μM carboplatin (1.42 ± 0.5), and 2 μM PFOA + 400 μM carboplatin (1.36 ± 0.28). Interestingly, only HEC-1 cells exposed to 2 μM PFHpA then treated with 25 μM cisplatin displayed increased survival fraction, suggesting PFAS exposure has a greater effect on HEC-1 cell carboplatin sensitivity compared to that of cisplatin. In Ishikawa cells, survival fraction did not increase compared to the vehicle control after PFAS exposure and treatment with either chemotherapeutic (Figure S6).

To determine the effects of PFAS mixtures on response to platinum-based chemotherapies in endometrial cancer cells, HEC-1 and Ishikawa cells were exposed to 1:1 mixtures of PFOA + PFHpA, PFOA + PFPA, PFHpA + PFPA, or a 1:1:1 mixture of PFOA + PFHpA + PFPA. Since these mixtures were sub-cytotoxic at all concentrations tested (Figures S7 and S8), 1 μM + 1 μM was selected for 1:1 mixtures of 2 PFAS chemicals (referred to as PFOA + PFHpA, PFOA + PFPA, and PFHpA + PFPA) while 0.75 μM + 0.75 μM + 0.75 μM was selected for the 1:1:1 mixture of all 3 chemicals (referred to as PFOA + PFHpA + PFPA). Interestingly, exposure to PFAS mixtures did not lead to increased survival fraction compared to the vehicle control in either HEC-1 or Ishikawa cells after treatment with platinum-based agents (Figures S9 and 10). Although no increases were observed, survival fraction significantly decreased compared to the vehicle control in Ishikawa cells exposed to PFAS mixtures then treated with cisplatin (Fig. 3). Specifically, survival fraction decreased significantly in Ishikawa cells exposed to PFHpA + PFPA + 10—50 μM cisplatin (largest decrease observed at 25 μM: 0.79 ± 0.13), PFOA + PFPA + 50 μM cisplatin (0.82 ± 0.18), and PFOA + PFHpA + PFPA + 10 μM cisplatin (0.82 ± 0.07). Although significant decreases in survival fraction were not observed for HEC-1 cells, trends towards a decrease were observed in PFHpA + PFPA-exposed cells treated with carboplatin and PFOA + PFHpA or PFOA + PFPA-exposed cells treated with cisplatin (Figure S9). Together, these findings demonstrate that exposure to PFAS mixtures decreases survival fraction in endometrial cancer cells, especially Ishikawa, treated with cisplatin. It is important to note that responses of HEC-1 and Ishikawa cells to carboplatin versus cisplatin treatment differed significantly by cell line and by chemotherapeutic used. In both cell lines, carboplatin efficacy may be influenced by environmental toxicants since platinum resistance was observed in PFAS-treated HEC-1 cells. Conversely, cisplatin appears to be more effective at reducing cell survival fraction in endometrial cancer cell lines, regardless of PFAS exposure.

### Effects of PFAS exposure and/or platinum-based chemotherapy treatment on mitochondrial membrane potential (ΔΨ_m_) in HEC-1 and Ishikawa cells

To better understand the mechanistic factors that contribute to the observed altered response to platinum-based chemotherapies, ΔΨ_m_ was evaluated in HEC-1 and Ishikawa cells post-PFAS exposure ± treatment with carboplatin or cisplatin. ΔΨ_m_ was measured by incubating endometrial cancer cells with 10 μg/mL JC-1 dye prior to exposure to PFAS followed by treatment with either carboplatin or cisplatin for 1 h. CCCP, a potent uncoupler of mitochondrial oxidative phosphorylation, was used as a positive control for the JC-1 assay.

Prior to investigating the effects of PFAS exposure ± platinum-based chemotherapy treatment on ΔΨ_m,_ the effects of carboplatin or cisplatin treatment alone were evaluated in HEC-1 and Ishikawa cells (Fig. 4a-e). In our previous study, ΔΨ_m_ decreased post-carboplatin treatment alone in platinum-sensitive ovarian cancer cells, likely indicative of cells undergoing apoptosis [32]. A similar trend was observed in endometrial cancer cell lines exposed to platinum-based chemotherapy, further suggesting their sensitivity to these agents in the absence of PFAS. For example, ΔΨ_m_ decreased by ~ 50% in HEC-1 cells (Fig. 4b) exposed to 50–400 μM carboplatin (0.56 ± 0.11). ΔΨ_m_ also decreased by ~ 79% in HEC-1 cells exposed to 100 μM CCCP (0.21 ± 0.02). Compared to that of carboplatin-treated HEC-1 cells, decreases in ΔΨ_m_ in carboplatin-treated Ishikawa cells, although significant, were less pronounced (Fig. 4c). The largest decrease was observed at 50 μM (0.67 ± 0.06), with only an 11% decrease being observed at 400 μM (0.88 ± 0.08). Additionally, 100 μM CCCP appeared less potent, as ΔΨ_m_ was only decreased by 36% (0.64 ± 0.17). In HEC-1 cells exposed to cisplatin (Fig. 4d), ΔΨ_m_ decreased at all doses by 30–40%, with the largest decrease being observed at 2.5 μM (0.61 ± 0.11). While ΔΨ_m_ significantly decreased in carboplatin-treated Ishikawa cells, no significant changes in ΔΨ_m_ were observed in cisplatin-treated Ishikawa cells (Fig. 4e). In fact, although insignificant, ΔΨ_m_ increased by 10% on average.

Interestingly, while most chemotherapy exposures decreased ΔΨ_m_, 1 h of PFAS exposure significantly increased ΔΨ_m_ in HEC-1 and Ishikawa cells (Fig. 4f-g). Although all PFAS-exposed HEC-1 cells were trending towards a significant increase in ΔΨ_m_, only four groups were significant: 2 μM PFOA, 0.5 μM PFHpA, 2 μM PFHpA, and 2 μM PFPA. ΔΨ_m_ increased most in the 2 μM PFHpA group (1.73 ± 0.16). Significantly increased ΔΨ_m_ was also observed in Ishikawa cells exposed to 2 μM PFHpA, 0.5 μM PFPA, and 2 μM PFPA. Increases in ΔΨ_m_ for PFAS-exposed Ishikawa cells were similar, with 2 μM PFHpA also leading to the largest increase (1.41 ± 0.39). No significant alterations in ΔΨ_m_ were observed in HEC-1 (Fig. 4h) cells following exposure to PFAS mixtures; however, Ishikawa cells exposed to PFHpA + PFPA or PFOA + PFHpA + PFPA displayed increased ΔΨ_m_ (Fig. 4i).

To determine the effect of concurrent PFAS exposure and platinum-based chemotherapy treatment, HEC-1 and Ishikawa cells were exposed to JC-1 dye prior to a simultaneous exposure to PFAS and either carboplatin or cisplatin. Interestingly, even in groups where altered survival fraction post-chemotherapy treatment was not observed, a significant increase in ΔΨ_m_ was seen (Figs. 5, S11a, S12a). For example, in HEC-1 cells exposed to PFOA, ΔΨ_m_ increased significantly in the 0.5 μM group with 400 μM carboplatin treatment and in the 2 μM group at all carboplatin levels by 40—50% (Fig. 5b). HEC-1 ΔΨ_m_ also increased in cells exposed to 0.5 μM PFHpA and 2 μM PFHpA at all carboplatin levels (Fig. 5d) by as much as 60% and 76%, respectively. The largest increase occurred in HEC-1 cells exposed to 2 μM PFHpA and treated with 200 μM carboplatin (1.76 ± 0.21). No significant increases in ΔΨ_m_ were observed in HEC-1 cells post-PFPA exposure and chemotherapy treatment (Fig. 5f). In Ishikawa cells, no changes in ΔΨ_m_ were observed in either PFOA group after carboplatin treatment (Fig. 5c). Increases in ΔΨ_m_ were observed in the 0.5 μM PFHpA group at nearly all levels by as much as 35% (Fig. 5e), 2 μM PFHpA group at all levels by as much as 46% (Fig. 5e), and 2 μM PFPA + 50 μM carboplatin by 34% (Fig. 5g).

Following exposure to PFAS mixtures, ΔΨ_m_ significantly increased in HEC-1 cells exposed to PFOA + PFHpA + PFPA at 50 μM, 100 μM, and 400 μM carboplatin by as much as 37% (Fig. 5h). Compared to HEC-1 cells, more instances of increased ΔΨ_m_ were observed after exposure to PFAS mixtures in Ishikawa cells (Fig. 5i). While ΔΨ_m_ was unchanged after exposure to PFOA + PFHpA and PFOA + PFPA + 0 – 800 μM carboplatin, ΔΨ_m_ was significantly increased after exposure to PFHpA + PFPA + 0 – 800 μM carboplatin by 27–38%, and PFOA + PFHpA + PFPA + 0 – 800 μM carboplatin by 34–45%. While trends in ΔΨ_m_ were similar within PFAS mixture exposure groups, the largest increase in ΔΨ_m_ was observed in cells exposed to PFOA + PFHpA + PFPA and treated with the highest dose of carboplatin (1.42 ± 0.27). Altogether, these findings demonstrate that ΔΨ_m_ significantly increased post-PFAS and PFAS mixture exposure in HEC-1 and Ishikawa cells, and decreased post-platinum-based chemotherapy treatment in the absence of PFAS.

The response of the PFAS-exposed cell lines to cisplatin was quite different from that of carboplatin for the ΔΨ_m_ endpoint. While PFAS exposure + carboplatin treatment led to significant increases in ΔΨ_m_ in HEC-1 cells, no significant changes in ΔΨ_m_ were seen after exposure to PFAS (Fig. 6b,d,f) or PFAS mixtures (Fig. 6h) concurrent with cisplatin treatment (Figure S11b). A trend towards increased ΔΨ_m_ was observed in several PFHpA exposure groups (Fig. 6d), but none were significantly different from controls. Compared to HEC-1 cells, more instances of increased ΔΨ_m_ were observed in Ishikawa cells after exposure to PFAS or PFAS mixtures and concurrent treatment with cisplatin (Figure S12b). Specifically, ΔΨ_m_ increased in the following exposure groups and treatment levels: 2 μM PFOA + 50 μM cisplatin (Fig. 6c, 25%), 0.5 μM PFHpA + 0—50 μM cisplatin (Fig. 6e, 26—46%), 2 μM PFHpA + 0 – 50 μM cisplatin (Fig. 6e, 21—38%), PFHpA + PFPA + 50 μM cisplatin (Fig. 6i, 29%), and PFOA + PFHpA + PFPA + 50 μM cisplatin (Fig. 6i, 28%). Trends in ΔΨ_m_ were similar across groups, but the largest increase in ΔΨ_m_ was observed in cells exposed to 0.5 μM PFHpA then treated with 50 μM cisplatin (1.46 ± 0.2). No change in ΔΨ_m_ was observed in Ishikawa cells exposed to PFPA + cisplatin (Fig. 6g). Altogether these findings demonstrate that, even in the absence of platinum resistance, ΔΨ_m_ is increased after PFAS or PFAS mixture exposure and platinum-based chemotherapy treatment in HEC-1 and Ishikawa cells. This warrants further exploration into PFAS-induced mitochondrial changes in endometrial cancer in relation to disease progression and therapy response.

## Discussion

In previous studies, environmental contaminants have been linked to the increased risk and progression of endometrial cancer [44]. For example, bisphenol A (BPA), an endocrine-disrupting compound, has been shown to affect the estrogen cycle as well as estrogen and progesterone regulation [45, 46]. Other studies have linked BPA exposure in endometrial cancer cells, specifically Ishikawa, to increased Ki67, a marker of proliferation [47]. In addition to BPA, 2,2′,4,4′-tetrabromodiphenyl ether (BDE-47), a common polybrominated diphenyl ether (PBDE), has been shown to increase proliferation and the metastatic capacity of Ishikawa and HEC-1B cells [48]. In cells that were treated with BDE-47, responses to cisplatin and paclitaxel were also decreased, suggesting the potential to induce chemoresistance [48]. While various environmental contaminant classes have been evaluated in the context of endometrial cancer, PFAS have been minimally explored, and never in the context of chemotherapy resistance. In this work, we report, for what we believe is the first time, that PFAS and PFAS mixture exposure alters HEC-1 and Ishikawa cells response to platinum-based chemotherapies. PFOA, PFHpA, PFPA, and mixtures of these agents are present in many drinking water supplies and food sources worldwide, making these exposures extremely human-relevant with the potential to contribute to disease onset and outcomes.

Due to the association of select PFAS with endometrial disorders, it is plausible that PFAS exposure may influence the response to the platinum-based chemotherapeutics used in the treatment of recurrent or advanced-stage endometrial cancer. This represents a critical knowledge gap that is addressed by the present study, which evaluates the effects of globally-relevant PFAS, including the legacy molecule PFOA and emerging compounds PFHpA and PFPA, as well as mixtures of these chemicals on endometrial cancer survival fraction pre- and post-platinum-based chemotherapy treatment. Understanding the contribution of environmental toxicant exposure to chemotherapy response in endometrial cancer is critical for optimizing treatment regimens and improving survival. Here, we report that exposure to select PFAS, and mixtures, at sub-cytotoxic doses had differential effects on chemotherapy efficacy in the two endometrial cancer cell lines evaluated. HEC-1 survival fraction increased in cells exposed to certain PFAS then treated with carboplatin, indicative of platinum resistance, while minimal effects were observed after cisplatin treatment. Conversely, Ishikawa cells exposed to certain PFAS mixtures then treated with cisplatin displayed decreased survival fraction, indicative of an enhanced chemotherapeutic response, while no effects were observed after carboplatin treatment. This finding is interesting because, to our knowledge, this is the first instance of an enhanced chemotherapeutic response in PFAS-exposed cells, suggesting that the combination of insults tested here (i.e. exposure to sub-cytotoxic doses of certain PFAS mixtures followed by treatment with platinum-based chemotherapy) alter cellular function and compensatory mechanisms in a manner that the burden cannot be overcome. Additionally, while PFAS mixtures are more human relevant than individual agents, it is important to note that the mixtures used in this study contained a maximum of 3 PFAS chemicals. PFAS mixtures found in water supplies can contain tens of PFAS chemicals, thus exploring the impact of more complex PFAS mixtures is critical to understanding their true impact on chemotherapy response. These observations, along with our recently published findings [32], highlight the importance of devoting resources and developing a framework to broaden the scope of these studies to characterize the interaction between classes of PFAS and chemotherapeutic agents in a range of cancer cell lines. Regardless of survival fraction post-PFAS exposure + chemotherapy treatment, ΔΨ_m_ was increased in both cell lines, suggesting PFAS may target mitochondrial function in endometrial cancer cells. These findings are novel, as PFAS exposure in relation to chemotherapy response in endometrial cancer has never been explored, nor has the potential mechanism underlying these effects.

Since PFAS and PFAS mixtures are known to induce toxicity in several types of cell lines [49–51], the first aim of this study was to determine sub-cytotoxic concentrations of PFAS in endometrial cancer cell lines. Based on our previous study in ovarian cancer models [32], PFAS concentrations ranging from 0.025 – 2.25 μM were evaluated in HEC-1 and Ishikawa cells. Compared to other studies that have examined higher PFAS concentrations (ranging from micromolar to even millimolar amounts), the nanomolar and low micromolar concentrations used in the present study are more relevant to human exposure levels. In this study, all concentrations of PFOA, PFHpA, and PFPA tested (0.025 – 2 μM) were sub-cytotoxic, as evidenced by the fact that no significant decreases in survival fraction were observed in either HEC-1 or Ishikawa cells. In fact, in several exposure groups significant increases in survival fraction were observed in HEC-1 and Ishikawa cells. These findings agree with previous studies reporting increased survival or proliferation from PFAS exposure in human granulosa and breast epithelial cells [52–54]. Other studies have reported that PFOA induces increased migration and invasion in Ishikawa cells by activating ERK/mTOR pathway signaling [55]. Interestingly, in HEC-1 and Ishikawa cells, all concentrations of mixtures examined (0.3– 2.25 μM) were neither cytotoxic nor did they improve survival fraction. These findings differ compared to those observed in ovarian cancer cell lines, where significantly increased survival fractions following PFAS mixture exposures were observed [32]. Altogether, findings from the present study suggest that individual PFAS chemicals, but not mixtures, increase survival fraction in HEC-1 and Ishikawa cells, warranting further investigation into mechanisms underlying those potential proliferative effects.

Importantly, there are several epidemiologic studies reporting serum PFAS concentrations in women with endometriosis [9, 10, 13, 56]. Although endometriosis is not directly related to endometrial cancer or chemoresistance related to the disease, these concentrations may be most relevant as they are already correlated with adverse health effects relating to endometrial tissue. Hammarstand et al. [56] evaluated serum PFAS concentrations in women living near Ronneby, Sweden and found that in those with self-reported endometriosis, geometric mean levels of PFOA, PFOS, and PFNA were elevated compared to women not reporting endometriosis and ranged from 1—16 ng/mL. In a 2003 – 2006 National Health and Nutrition Examination Survey (NHANES) study evaluating PFAS serum concentrations in women, Campbell et al. [9] found that women with endometriosis had significantly elevated serum concentrations of PFOA (3.48 ng/mL vs. 2.84 ng/mL), PFOS (16.28 ng/mL vs. 13.36 ng/mL), and PFNA (1 ng/mL vs. 0.84 ng/mL) compared to women without endometriosis. Differences in serum concentration of individual PFAS chemicals may be explained by a variety of sociodemographic factors, including geographic location, sex, age, and race. Nonetheless, the range of PFAS concentrations (7 – 828 ng/mL) used in this study overlap with those reported in women with endometriosis, demonstrating the human relevance of the selected concentrations and their potential to impact female reproductive cancer outcomes.

Although the prognosis for patients with early-stage endometrial cancer is quite good, survival remains poor for patients with recurrent or advanced-stage disease [57]. In patients with localized disease, 5-year survival is ≳ 95%, compared to 17% in patients with distant metastases [22]. For patients with advanced-stage disease, chemotherapy regimens involving a combination of platinum-based compounds, such as carboplatin or cisplatin, or doxorubicin with taxane-based compounds, such as paclitaxel, may be used, with carboplatin/paclitaxel ± PD-1 inhibitor being the preferred standard of care treatment regimen [21, 24, 57–60]; however, many patients will develop chemoresistance [21, 26, 28]. In women who have previously responded to platinum-based treatment, ~ 40% or ≳ 60% of patients will respond to an additional cycle, depending on whether their platinum-free interval was < 12 months or > 12 months, respectively [18, 61, 62]. Response rates are < 20% for second-line chemotherapy treatment in patients with metastatic endometrial cancer [18, 63]. Thus, identifying factors that contribute to diminished chemotherapy response, such as environmental contaminant exposure, is critical to optimize treatment regimens and improve response rates.

Since the combination of platinum- and taxane-based agents are most common for the treatment of advanced-stage or recurrent endometrial cancer, this study explores, for the first time, the effect of PFAS exposure on the response of endometrial cancer cells to platinum-based chemotherapies. Carboplatin and cisplatin were evaluated because even though carboplatin is preferred due to lower toxicity [24, 64], cisplatin is one of the most effective agents for the treatment of endometrial cancer [26, 65]. In ovarian cancer cell lines, PFAS and PFAS mixture exposure has been shown to induce resistance to carboplatin [32]. In this study, increased survival fraction was also observed in HEC-1 cells exposed to PFAS, but not PFAS mixtures, and then treated with carboplatin. After exposure to 2 μM PFOA, 0.5 μM and 2 μM PFHpA, or 2 μM PFPA and treatment with 200 – 400 μM carboplatin, survival fraction significantly increased compared to controls. These findings, specifically that PFAS are more likely to induce platinum resistance at higher doses of carboplatin, are consistent with previous findings in ovarian cancer cell lines [32]. No instances of increased survival fraction were observed in Ishikawa cells exposed to PFAS or PFAS mixtures followed by treatment with carboplatin. After PFAS or PFAS mixture exposure and cisplatin treatment, HEC-1 cell survival fraction was unchanged compared to controls in most groups; however, a statistically significant increase was observed in 2 μM PFHpA-exposed cells treated with 25 μM cisplatin. Conversely, significant decreases in survival fraction were seen in Ishikawa cells exposed to PFAS mixtures and then treated with cisplatin. These findings differ from those reported in ovarian cancer cell lines, where select PFAS mixtures increased survival fraction after carboplatin exposure and no PFAS exposure groups led to decreased survival fraction in combination with platinum-based chemotherapy [32]. Nonetheless, the finding that PFAS exposure increases survival fraction significantly post-carboplatin treatment in HEC-1 cells is critical since carboplatin is the preferred platinum-based chemotherapeutic for the treatment of endometrial cancer [24, 64]. Despite the lowered survival fraction in Ishikawa cells exposed to PFAS mixtures then treated with cisplatin, toxicity concerns for cisplatin are greater than those of carboplatin, preventing justification for using cisplatin clinically in the treatment of endometrial cancer at this time. If future studies continue to illustrate lowered survival fractions in endometrial cancer cells exposed to PFAS mixtures then treated with cisplatin, it may be worth considering the use of cisplatin, perhaps at lower doses to mitigate excess toxicity, in select populations of PFAS mixture-exposed endometrial cancer patients.

The observed increase in ΔΨ_m_ in HEC-1 and Ishikawa cells post-PFAS exposure ± chemotherapy treatment suggests that mitochondrial changes are likely to play a role in altered chemotherapy response. Although treatment response after PFAS exposure differed by cell line and platinum-based agent, determining the precise mechanistic factors that contribute to the observed altered chemotherapy response is important. Since mitochondria have been implicated in chemoresistance and PFAS can disrupt mitochondrial function [50, 66–70], evaluating the effects of PFAS exposure on mitochondrial function in endometrial cancer cells may reveal a mechanism by which chemoresistance occurs. In ovarian cancer cell lines, altered mitochondrial function has been shown to underlie platinum resistance [40], and altered ΔΨ_m_ has been reported in PFAS-exposed and PFAS-induced platinum resistant cells [32]. Thus, ΔΨ_m_ was evaluated in HEC-1 and Ishikawa cells post-PFAS exposure alone and in combination with either carboplatin or cisplatin. ΔΨ_m_ is an indicator of mitochondrial health that can be measured using various commercially available dyes, including JC-1 and Rhodamine123 [71]. Measuring ΔΨ_m_ has limitations inherent to each dye, and these should be considered when selecting which dye to use as well as in interpretation of results. In this study, cells were treated with JC-1 dye prior to PFAS exposure and/or chemotherapy treatment. Compared to other ΔΨ_m_ dyes, the JC-1 dye is unique in that in unbound form, JC-1 molecules emit green fluorescence; however, the aggregates it forms when it accumulates in mitochondria, known as J-aggregates, fluoresce red [32, 72]. Therefore, the ratio of red to green fluorescence can indicate the polarization state of the mitochondrial membrane, which can indirectly inform the overall state of mitochondrial health [32, 72]. For example, in cells undergoing apoptosis, ΔΨ_m_ is typically decreased due to mitochondrial membrane depolarization. This often occurs in cells treated with chemotherapy [32, 73, 74], since the mode of action of many chemotherapeutics, in particular platinum-based compounds, is to generate reactive oxygen species-induced apoptosis. In HEC-1 cells exposed to carboplatin or cisplatin alone and Ishikawa cells exposed to carboplatin alone, ΔΨ_m_ was significantly decreased compared to controls, suggesting these cells were shifting towards undergoing apoptosis after chemotherapy exposure. Decreased ΔΨ_m_ in endometrial cancer cells has also been reported by others after treatment with histone deacetylase inhibitors [75] and a combination treatment of trichostatin A and paclitaxel [76]. Although ΔΨ_m_ appeared to increase in Ishikawa cells post-cisplatin treatment, which has not been reported previously, we suspect this effect may be due to short exposure time prior to JC-1 aggregate ratio measurement. Contrary to the responses post-chemotherapy treatment, ΔΨ_m_ was significantly increased after exposure to select concentrations of PFOA, PFHpA, PFPA, and/or select PFAS mixtures. While decreases in ΔΨ_m_ are associated with apoptosis, increases in ΔΨ_m_ may suggest enhanced bioenergetic signaling or adenosine triphosphate generating capacity [77] and are associated with enhanced invasive properties in cancer cells [78]. Additionally, cancer cells have been shown to have increased ΔΨ_m_, which may be attributed to elevated reactive oxygen species levels or biochemical signaling pathway activation [78]. Regardless of the mechanism underlying the increase in ΔΨ_m_ after exposure to PFAS, these findings differ from other studies evaluating ΔΨ_m_ after PFAS exposure, which have reported decreases in ΔΨ_m_ in human liver cells [68], pancreatic β cells [69], lymphocytes [79], and osteoblasts [66].

Since ΔΨ_m_ was increased after PFAS exposure alone in both cell lines, ΔΨ_m_ was evaluated in HEC-1 and Ishikawa cells post-chemotherapy treatment as well. Select PFAS exposures induced platinum resistance, specifically to carboplatin, in HEC-1 cells, thus increases in ΔΨ_m_ could suggest mitochondrial mechanisms underlying the observed resistance. In both cell lines, increases in ΔΨ_m_ were observed after carboplatin treatment in nearly all PFHpA exposure groups. Less frequent instances were observed in HEC-1 cells exposed to PFOA or PFAS mixtures as well as Ishikawa cells exposed to PFPA or PFAS mixtures. This is similar to the previous report that PFAS-exposed and carboplatin-treated ovarian cancer cells displayed increased ΔΨ_m_ compared to controls [32]. No changes in ΔΨ_m_ were observed in HEC-1 cells exposed to PFAS then treated with cisplatin; however, instances of increased ΔΨ_m_ were observed in Ishikawa cells exposed to PFOA, PFHpA, PFHpA + PFPA, and PFOA + PFHpA + PFPA then treated with cisplatin. Increases in ΔΨ_m_ after PFAS mixture + cisplatin treatment in Ishikawa cells were unexpected, as these groups displayed decreased survival fractions compared to controls, indicative of an enhanced response to chemotherapy. A potential explanation for this discrepancy is that changes in ΔΨ_m_ after PFAS exposure may be transient. Initial increases in ΔΨ_m_ that may occur due to PFAS exposure can potentially be reduced over time by continued treatment with chemotherapy. Another potential explanation for this effect is the difference in treatment protocols for these experiments. In ΔΨ_m_ experiments, cells are co-incubated with PFAS + chemotherapy, whereas in PFAS + chemotherapy response studies, cells are exposed to PFAS for 48 h then treated with chemotherapy for 48 h in fresh medium. Since changes in ΔΨ_m_ were observed in cells with PFAS-induced platinum resistance (HEC-1—carboplatin) and PFAS mixture-induced platinum sensitivity (Ishikawa – cisplatin), further investigation into the time-dependent effects of PFAS exposure on mitochondrial function is critical.

It is important to note that ΔΨ_m_ is a highly time-sensitive and transient endpoint to measure. Based on optimization experiments performed in ovarian cancer cells, 1-h appeared to be the optimal endpoint for the JC-1 readout, compared to immediate, 30-min, or 2-h exposures. The goal of the 1-h JC-1 assay is to understand how ΔΨ_m_ is affected in the brief presence of a chemotherapeutic agent, which can suggest cell growth or apoptosis. In our previous study, and in this one, ΔΨ_m_ is reduced by as much as 70% after just a one-hour incubation with platinum-based agents. This suggests that the cells are undergoing apoptosis even after this short amount of time, thus confirming the 1-h time point as effective for this assay. Unfortunately, there is no way to know whether this amount of time is human relevant since ΔΨ_m_ cannot be measured in vivo in humans.

Although studies evaluating ΔΨ_m_, as it relates to chemoresistance in endometrial cancer, are lacking, several factors known to induce chemoresistance in endometrial cancer are also affected by PFAS exposure and should be explored in future studies. For example, exposure of endometrial cancer cells to estrogen can induce chemoresistance through activation of GRP78, a glucose-regulated protein [26, 57, 80]. PFAS have also been shown to mimic estrogens and can influence estrogen receptor activity directly or indirectly [81–83]. Additionally, PI3K/AKT pathway activation has been linked to chemoresistance in endometrial cancer [57, 84–86]. Numerous studies have shown that PFAS exposure in various types of cells induces migration and invasion through activation of the PI3K/AKT pathway [55, 87, 88]. In addition to mitochondrial endpoints, the effects of PFAS exposure on estrogen receptors and regulated pathways, including PI3K/AKT in HEC-1 and Ishikawa cells should be examined to understand mechanisms underlying platinum-based chemoresistance. Understanding the effects of PFAS on these pathways is critical since HEC-1 cells display mutations in KRAS and PIK3CA while Ishikawa cells have mutations in PIK3R1 and PTEN [89].

This is the first study to demonstrate that select PFAS and PFAS mixtures alter the response of endometrial cancer cell lines to platinum-based chemotherapy. Exposure to individual PFAS, but not PFAS mixtures, increased survival fraction in HEC-1 and Ishikawa cells prior to chemotherapy treatment. Additionally, PFAS exposure in HEC-1 cells, but not Ishikawa, led to significant increases in survival fraction after platinum-based chemotherapy treatment, indicative of platinum resistance. Conversely, Ishikawa cells exposed to PFAS mixtures then treated with cisplatin displayed significant decreases in survival fraction, suggesting that PFAS mixture exposure sensitizes cells to cisplatin. While both cell lines represent endometrioid endometrial cancer, they differ in proliferative and migratory capacities [90], as well as receptor expression [43], suggesting differential molecular profiles that may explain the variable response to PFAS exposure and chemotherapy treatment. Enhanced sensitivity to cisplatin may also result from increased potency of the compound compared to carboplatin, since cisplatin is known to be more systemically toxic [24, 64]. As a potential mechanism underlying alterations in chemotherapy response observed after PFAS exposure, ΔΨ_m_ was measured to understand the state of mitochondrial health. Interestingly, in HEC-1 cells, ΔΨ_m_ significantly increased after PFAS exposure and carboplatin treatment while in Ishikawa cells, ΔΨ_m_ increased significantly after PFAS exposure and treatment with either platinum-based agent. Findings from such experiments could shed light on precise mechanisms underlying the contribution of environmental stimuli to chemotherapy response in the context of endometrial cancer. Identifying such stimuli is critical for the development of personalized treatment regimens that would ultimately improve patient survival. Patients that may benefit from these findings are those living in PFAS-contaminated communities, as populations at risk for chemotherapy resistance based on serum PFAS profiles could be identified. If these patient populations could be identified prior to the administration of treatment, treatment failure, which is directly correlated with decreased survival, could be prevented.

### Supplementary Information

**Additional file 1.**

## Data Availability

All datasets supporting the conclusions of this articleare available on NIEHS’ CEBS data sharing platform: https://doi.org/10.22427/NTP-DATA-500-009-001-000-5

## Abbreviations

BDE-47: 2,2′,4,4′-Tetrabromodiphenyl ether
BPA: Bisphenol A
CCCP: Carbonyl cyanide m-chlorophenyl hydrazine
ECACC: European Collection of Authenticated Cell Cultures
FBS: Fetal bovine serum
HEPES: N-2-hydroxyethylpiperazine-N'-2-ethanesulfonic acid
JC-1: 5,5’,6,6’-tetrachloro-1,1′3,3’-tetraethylbenzimidazolocarbo-cyanine iodide
MEM/EBSS: Minimum Essential Medium with Earle’s Balanced Salt Solution
mtDNA: Mitochondrial deoxyribonucleic acid
NHANES: National Health and Nutrition Examination Survey
PBDE: Polybrominated diphenyl ether
PBS: Phosphate buffered saline
PFAS: Per- and poly-fluoroalkyl substances
PFBS: Perfluorobutane sulfonic acid
PFHpA: Perfluoroheptanoic acid
PFNA: Perfluorononanoic acid
PFOA: Perfluorooctanoic acid
PFOS: Perfluorooctane sulfonate
PFPA: Perfluoropentanoic acid
SD: Standard deviation
ΔΨm: Mitochondrial membrane potential
