# Supplementary material for: Exposure to select PFAS and PFAS mixtures alters response to platinum-based chemotherapy in endometrial cancer cell lines
Source: Environ Health. 2023 Dec 14;22:87. doi: 10.1186/s12940-023-01034-2 (PMC10720226; doi:10.1186/s12940-023-01034-2)
Supplement: Supplementary file 1 — Additional file 1. [file 12940_2023_1034_MOESM1_ESM.docx]

**
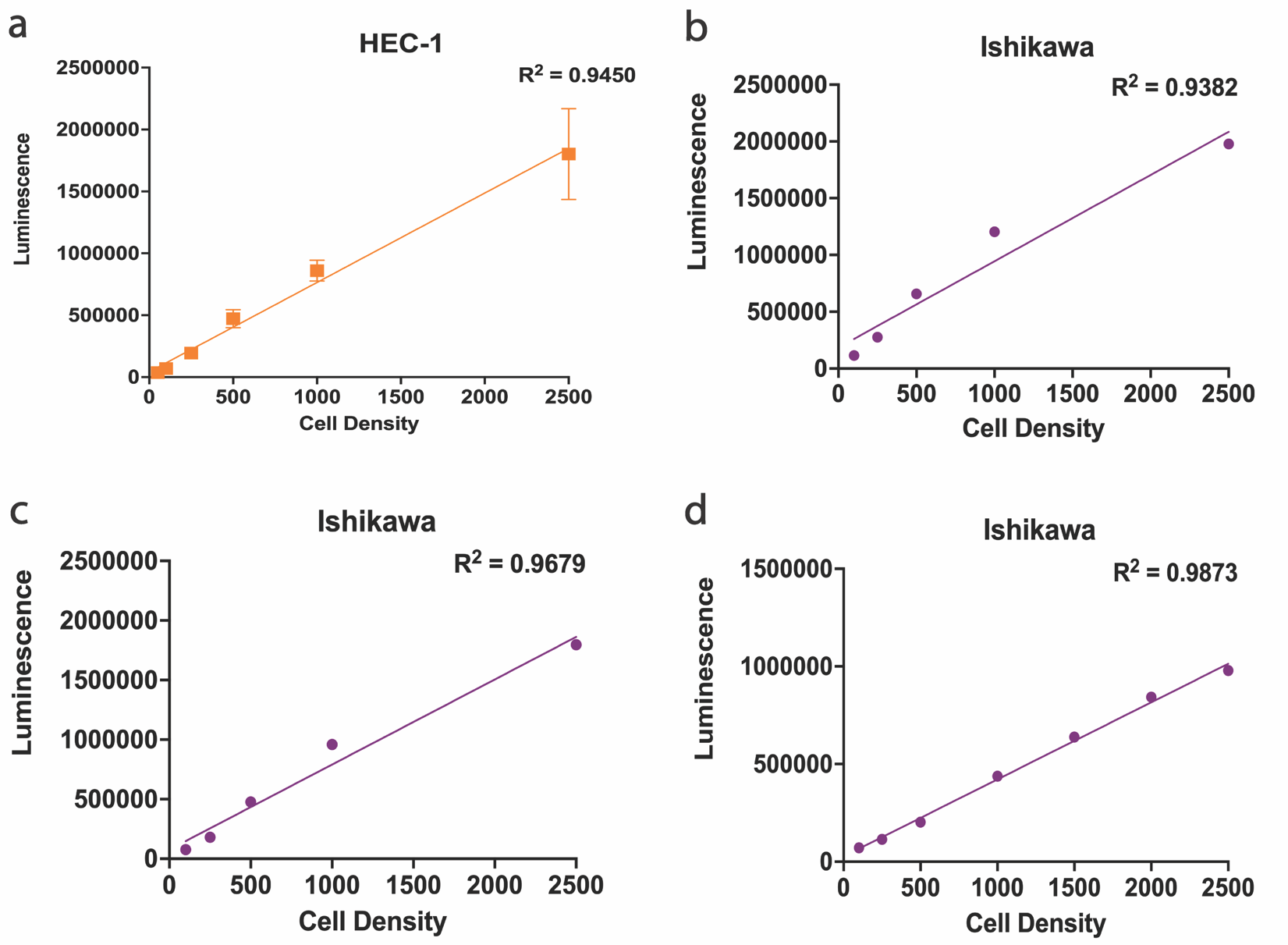
Figure S1**: Optimal seeding densities of HEC-1 and Ishikawa cells based on the linear dynamic range of the CellTiter Glo Luminescent Cell Viability Assay. (a) HEC-1 and (b-d) Ishikawa cells were plated at cell densities ranging from 0 - 2,500 cells/well and luminescence was read after 6 days. Data shown are mean ± standard deviation (SD); n=2 independent experiments in duplicate for HEC-1, n=3 independent experiments in duplicate for Ishikawa. Ishikawa shown as 3 separate graphs due to varying luminescence values.

**
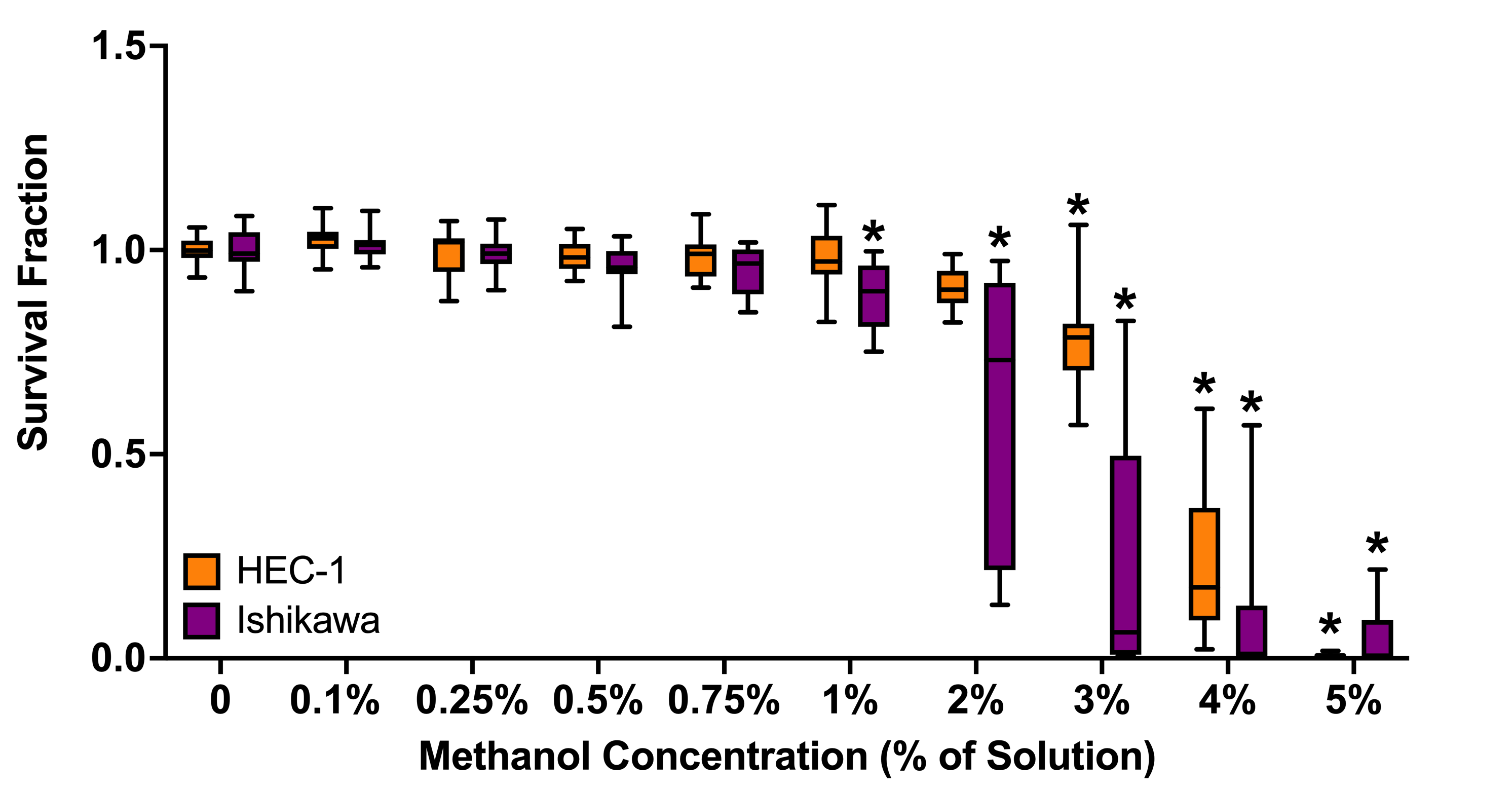
Figure S2**: HEC-1 and Ishikawa survival fraction post-methanol exposure. In both HEC-1 and Ishikawa cells, significant decreases in survival fraction were observed at methanol concentrations of 2 - 5%. No significant reductions in survival fraction were observed at concentrations < 1% methanol. Data are expressed as a percentage of the no methanol control (dashed line); n=3-4 independent experiments in sextuplicate. Significant differences between methanol exposure groups versus the untreated control are denoted by * (*p* < 0.05).

**
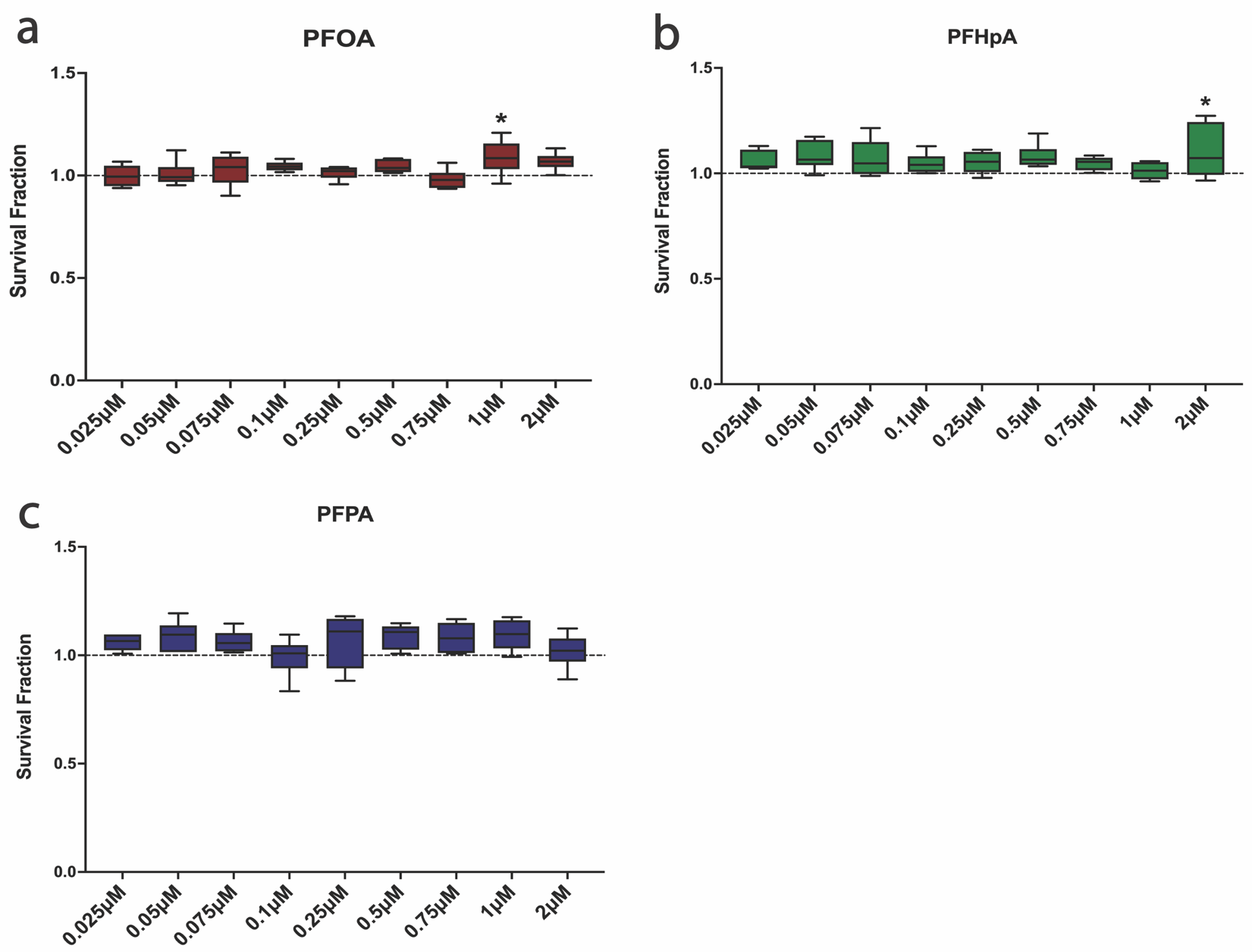
Figure S3**: Select nanomolar and micromolar concentrations of PFAS are sub-cytotoxic in HEC-1 cells. At concentrations ranging from 0.025 – 2 μM, (a) PFOA, (b) PFHpA, and (c) PFPA were sub-cytotoxic in HEC-1 cells. Select exposures, such as 1 μM PFOA (1.088 ± 0.083) and 2 μM PFHpA (1.103 ± 0.125) significantly increased survival fraction compared to the vehicle control (dashed line). Data shown are normalized to the vehicle control; n=3 independent experiments in duplicate. Significant differences between PFAS exposure groups versus the vehicle control are denoted by * (*p* < 0.05).

**
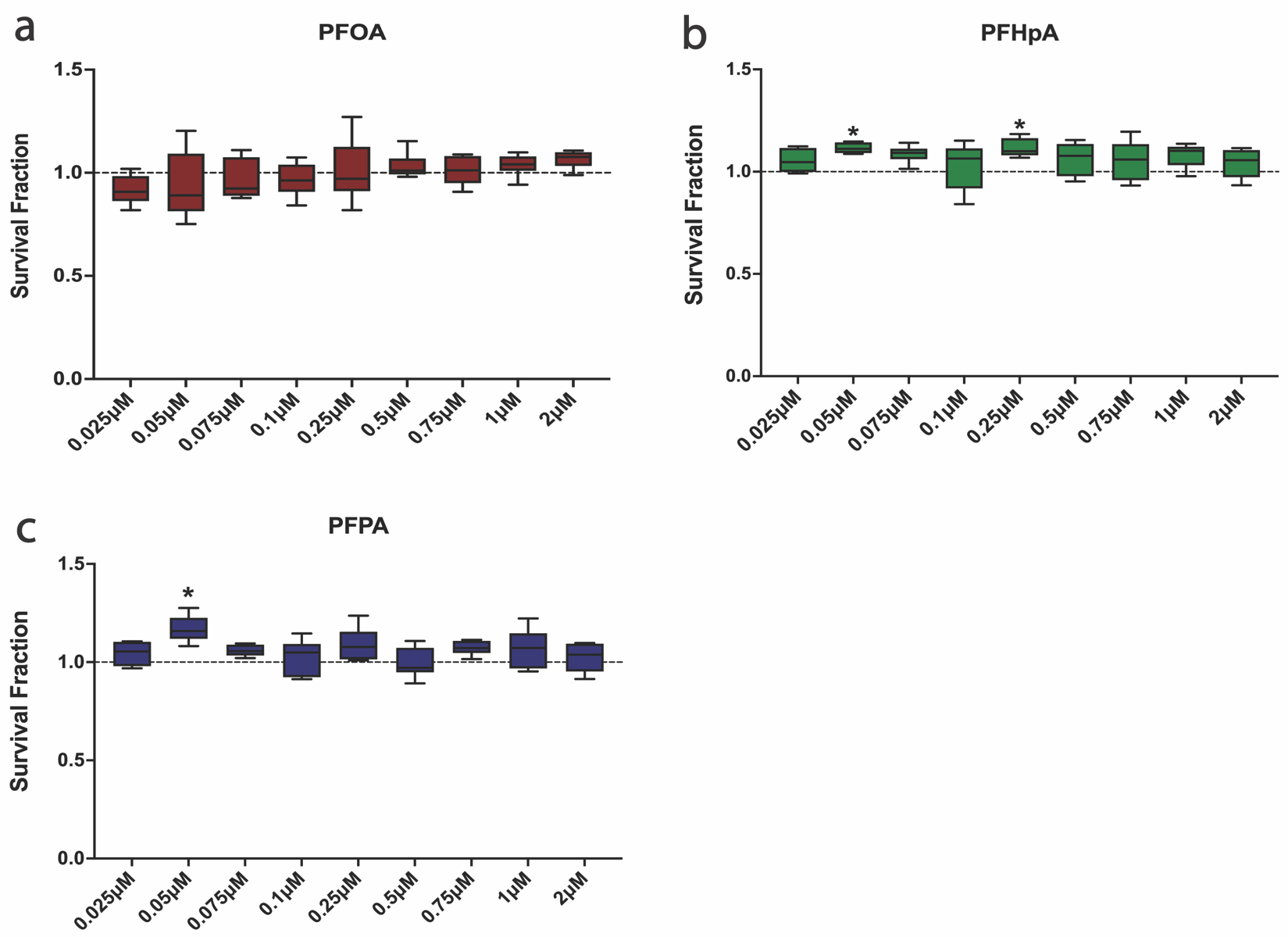
**

**Figure S4**: Select nanomolar and micromolar concentrations of PFAS are sub-cytotoxic in Ishikawa cells. At concentrations ranging from 0.025 – 2 μM, (a) PFOA, (b) PFHpA, and (c) PFPA were sub-cytotoxic in Ishikawa cells. Exposure to 0.05 μM PFHpA (1.115 ± 0.025), 0.25 μM PFHpA (1.116 ± 0.045), and 0.05 μM PFPA (1.169 ± 0.067) significantly increased survival fraction compared to the vehicle control (dashed line). Data shown are normalized to the vehicle control; n= at least 2 independent experiments in duplicate. Significant differences between PFAS exposure groups versus the vehicle control are denoted by * (*p* < 0.05).

**
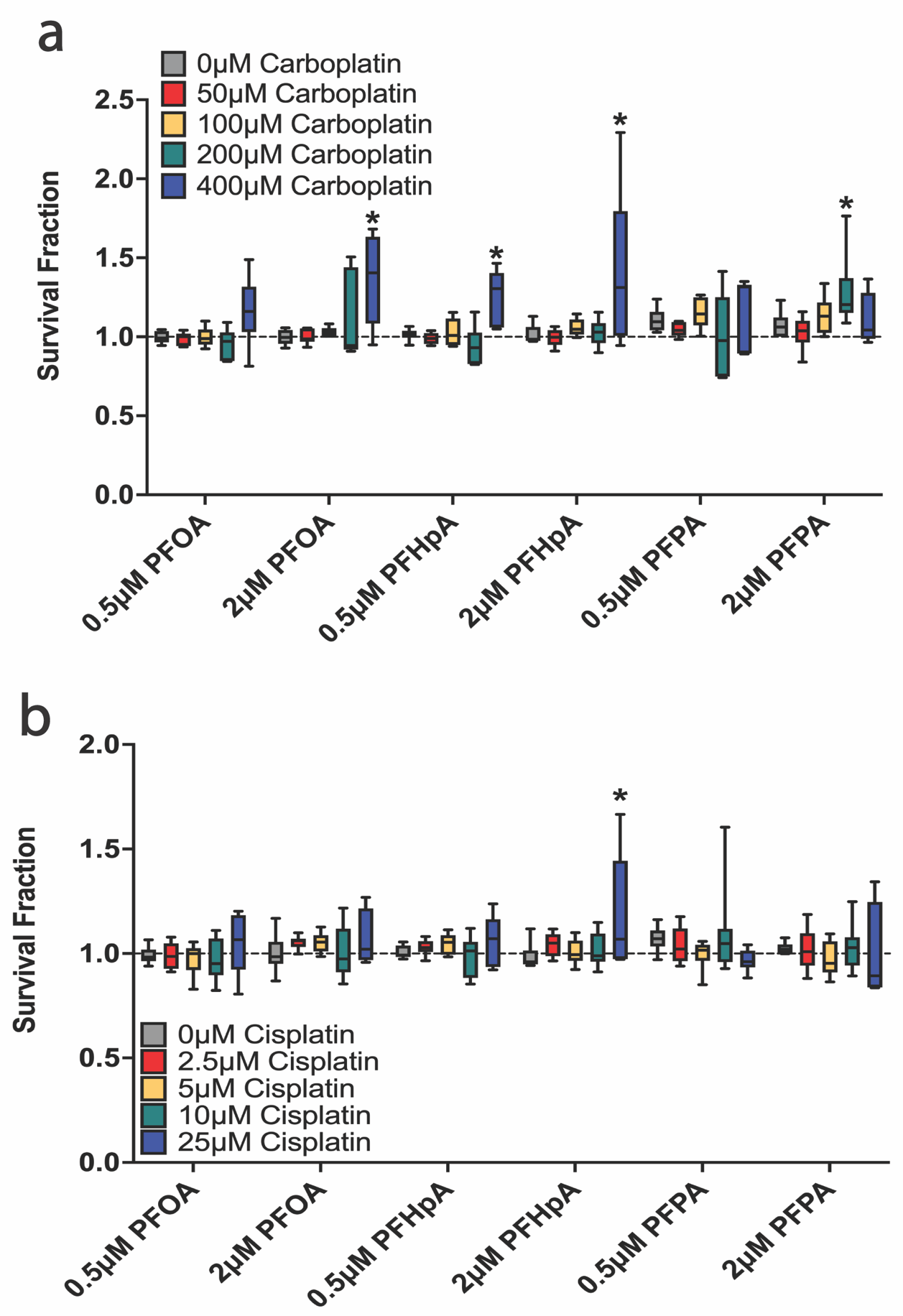
**

**Figure S5**: Survival fraction increased after PFAS exposure + platinum-based chemotherapy treatment in HEC-1 cells. (a) In HEC-1 cells exposed to PFAS + carboplatin, survival fraction significantly increased compared to the vehicle control (dashed line) in the 2 μM PFOA + 400 μM carboplatin (1.364 ± 0.282), 0.5 μM PFHpA + 400 μM carboplatin (1.262 ± 0.171), 2 μM PFHpA + 400 μM carboplatin (1.419 ± 0.502), and 2 μM PFPA + 200 μM (1.279 ± 0.243) carboplatin groups. Survival fraction also significantly increased in 500 nM PFPA-exposed cells without carboplatin. (b) Survival fraction was unchanged in HEC-1 cells exposed to PFAS then treated with cisplatin. Data shown are normalized to the vehicle control; n= at least 3 independent experiments in duplicate. Significant differences between PFAS + chemotherapy treatment group versus vehicle group at each respective chemotherapy dose are denoted by * (*p* < 0.05).


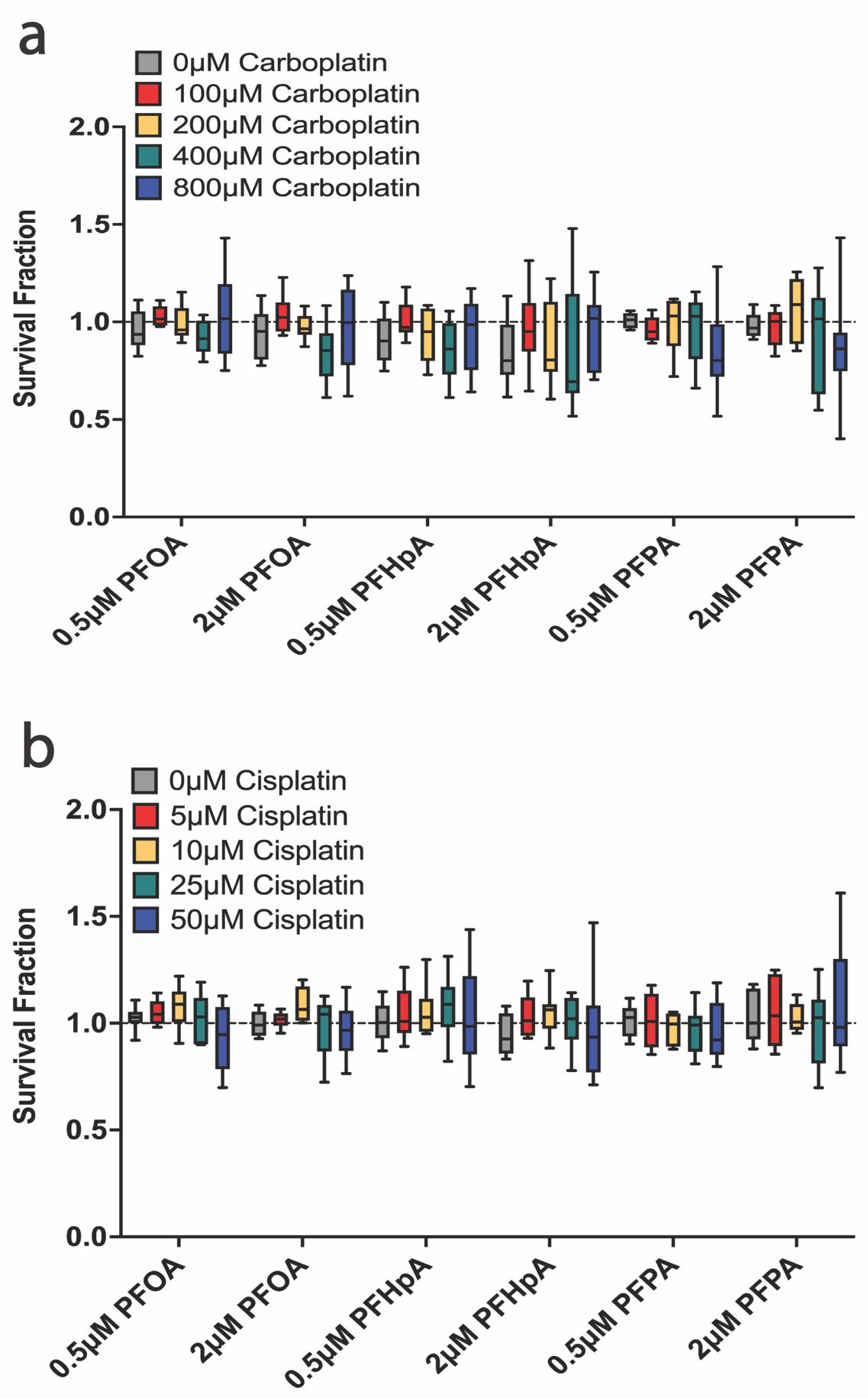


**Figure S6**: Survival fraction was unchanged in Ishikawa cells exposed to PFAS then treated with platinum-based chemotherapy. (a) In Ishikawa cells exposed to PFAS + carboplatin, survival fraction was not significantly increased compared to the vehicle control (dashed line) in any exposure group. (b) Survival fraction was also unaltered in Ishikawa cells exposed to PFAS + cisplatin in all exposure groups. Data shown are normalized to the vehicle control; n=4 independent experiments in duplicate. Significant differences between PFAS + chemotherapy treatment group versus vehicle group at each respective chemotherapy dose are denoted by * (*p* < 0.05).


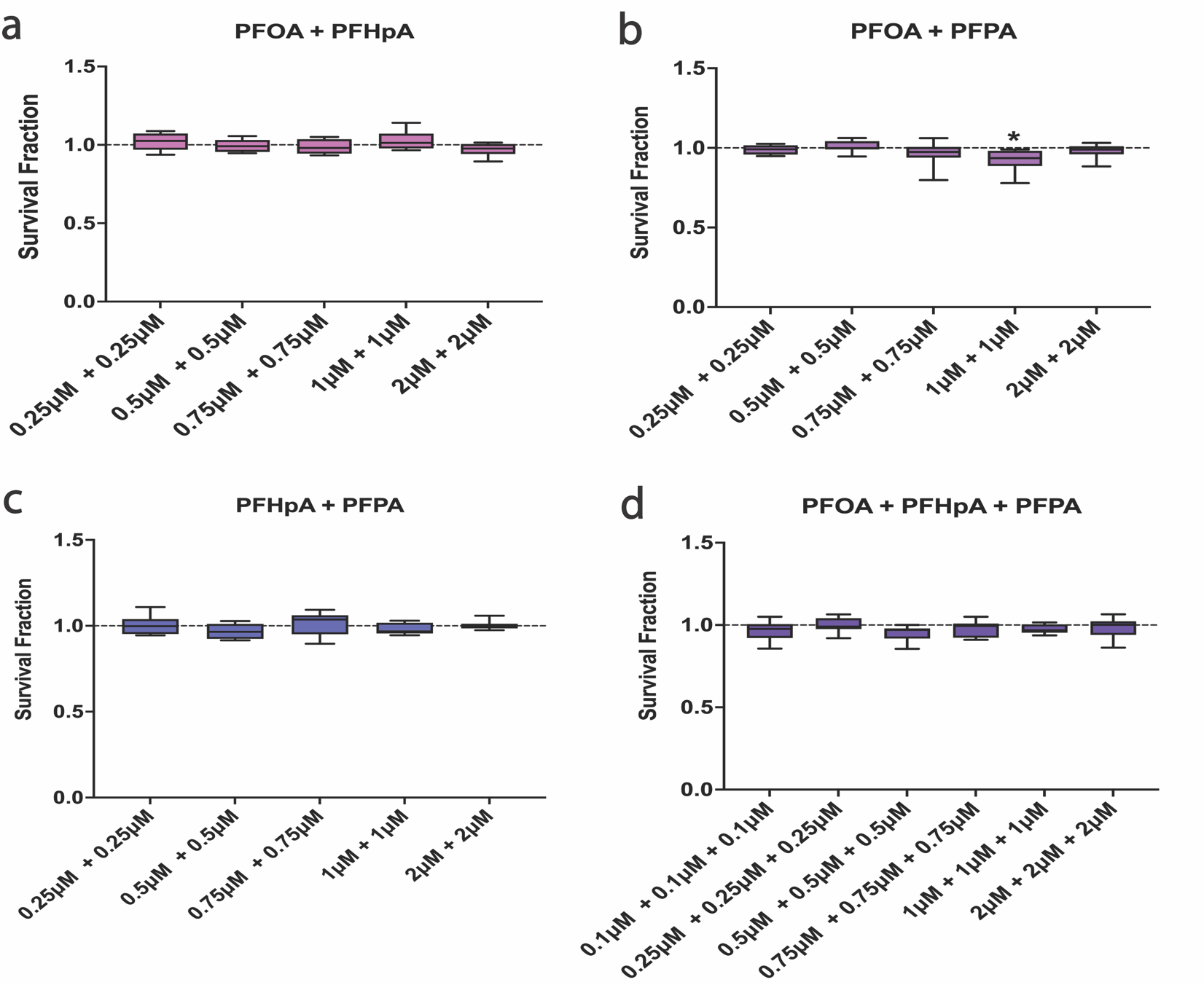


**Figure S7**: Select nanomolar and micromolar concentrations of PFAS mixtures are sub-cytotoxic in HEC-1 cells. Survival fraction was unchanged compared to the vehicle control (dashed line) in (a) PFOA + PFHpA-, (b) PFOA + PFPA-, (c) PFHpA + PFPA-, and PFOA + PFHpA + PFPA-exposed cells. Data shown are normalized to the vehicle control; n=3 independent experiments in at least triplicate.

**
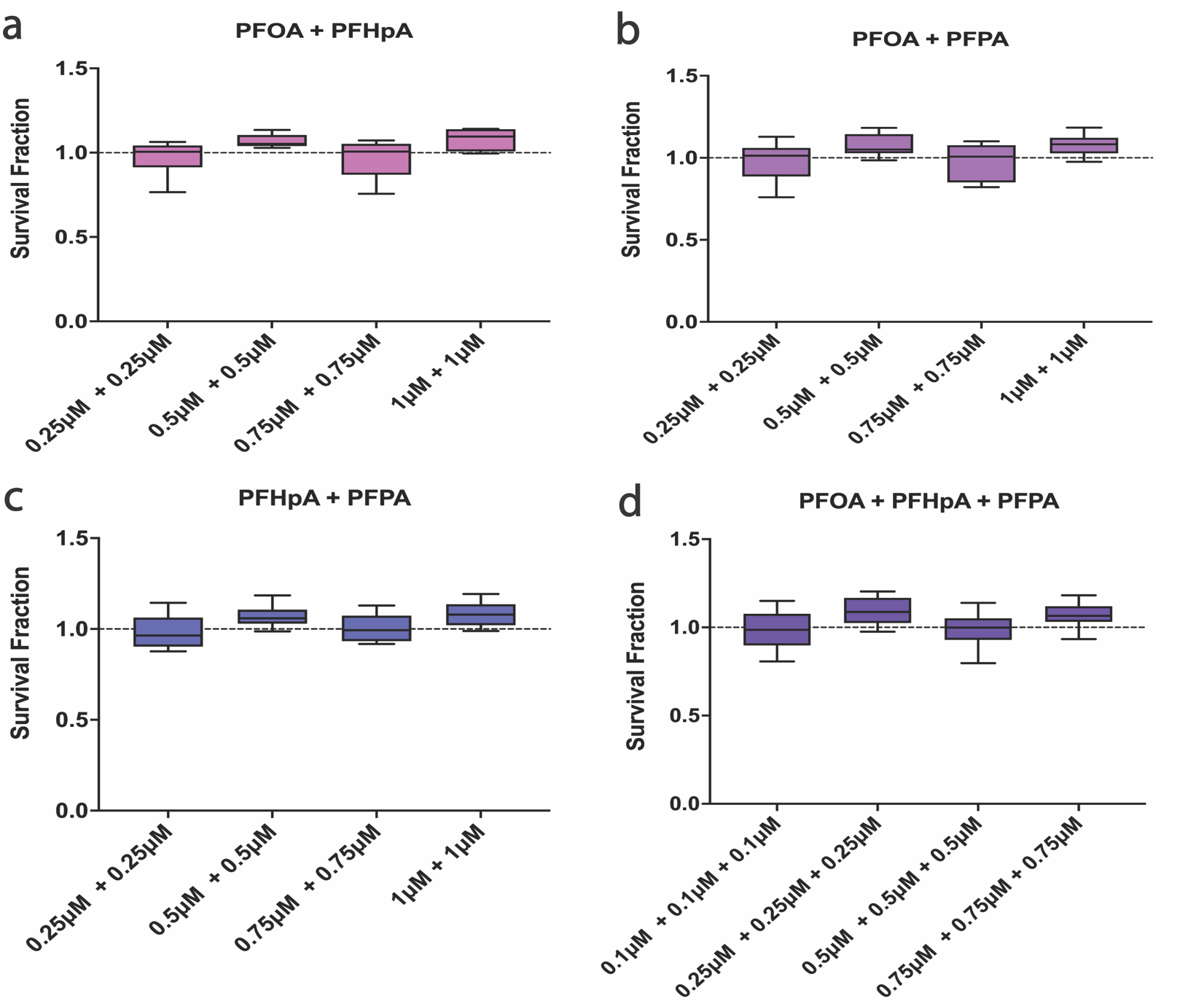
**

**Figure S8**: Select nanomolar and micromolar concentrations of PFAS mixtures are sub-cytotoxic in Ishikawa cells. Survival fraction was unchanged compared to the vehicle control (dashed line) in (a) PFOA + PFHpA-, (b) PFOA + PFPA-, (c) PFHpA + PFPA-, and PFOA + PFHpA + PFPA-exposed cells. Data shown are normalized to the vehicle control; n=3 independent experiments in triplicate.


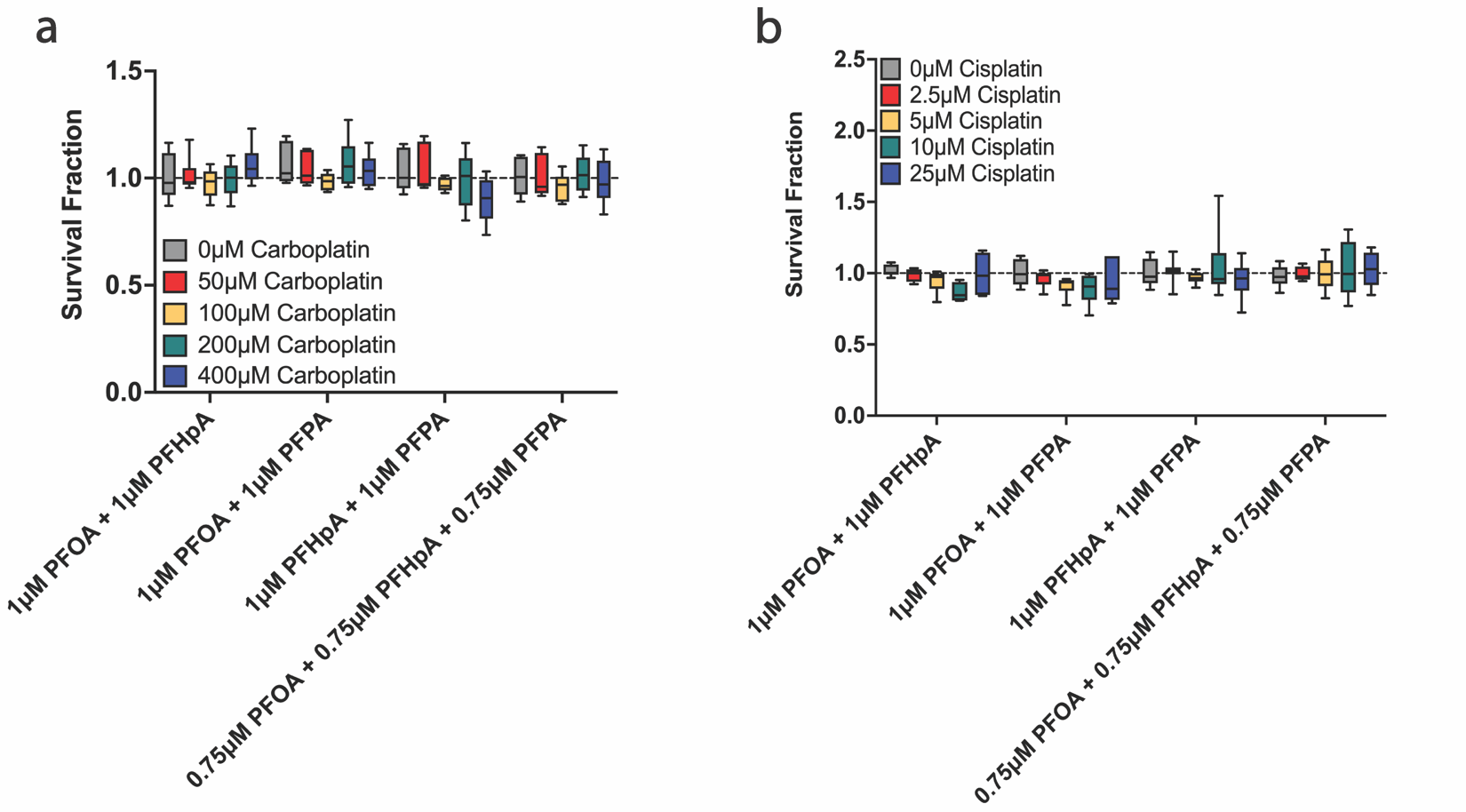


**Figure S9**: Survival fraction was unchanged in HEC-1 cells exposed to PFAS mixtures then treated with platinum-based chemotherapy. (a) In HEC-1 cells exposed to PFAS mixtures + carboplatin, survival fraction was not significantly increased compared to the vehicle control (dashed line) in any exposure group. (b) Survival fraction was also unchanged in HEC-1 cells exposed to PFAS mixtures + cisplatin in all groups. Data shown are normalized to the vehicle control; n= at least 3 independent experiments in duplicate.


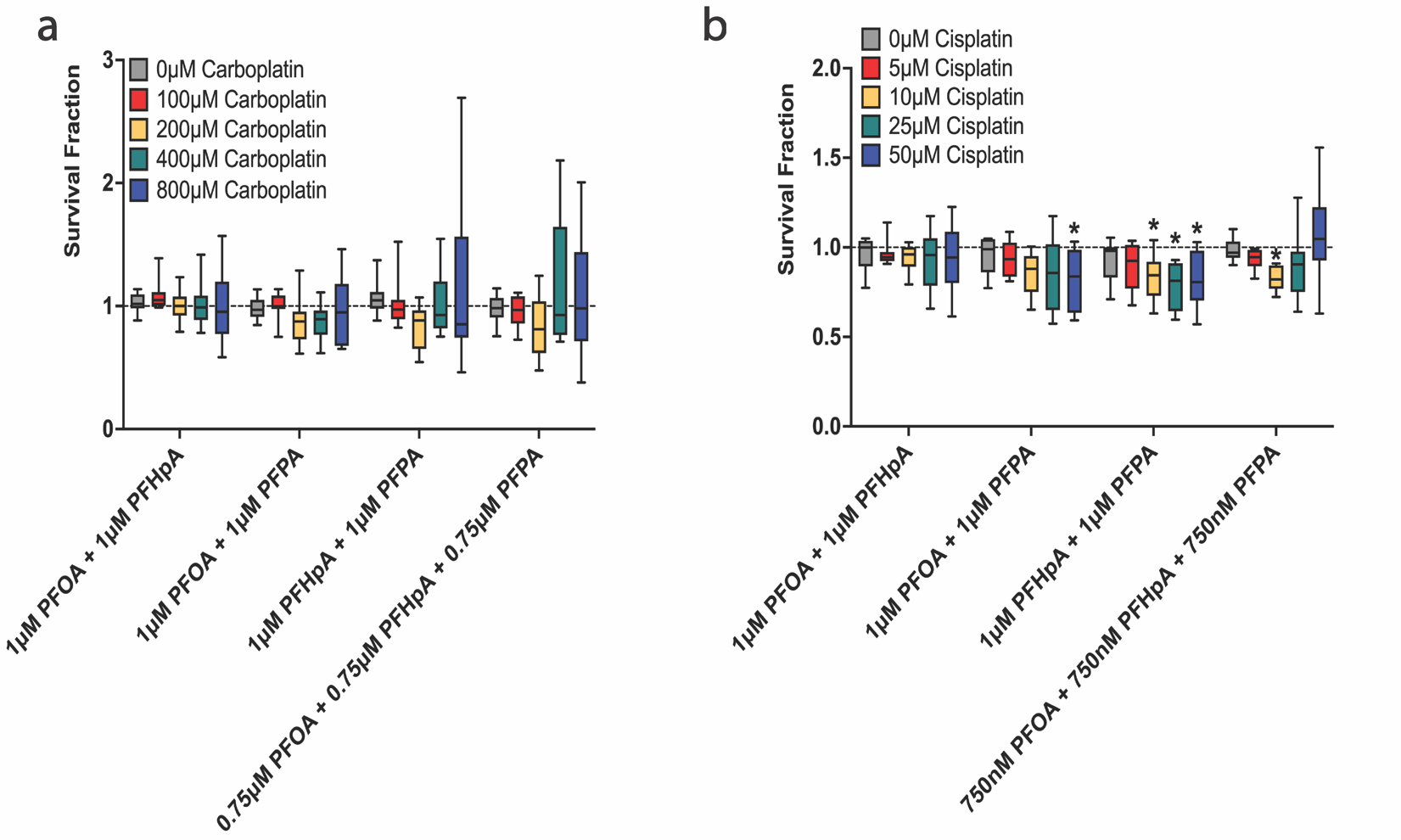


**Figure S10**: Survival fraction decreased after PFAS mixture exposure + cisplatin, but not carboplatin, treatment in Ishikawa cells. (a) No changes in survival fraction were observed in Ishikawa cells exposed to PFAS then treated with carboplatin. (b) Significant decreases in survival fraction compared to the vehicle control (dashed line) were observed in Ishikawa cells exposed to 1 μM PFOA + 1 μM PFPA + 10 μM cisplatin (0.857 ± 0.124), 1 μM PFOA + 1 μM PFPA + 50 μM cisplatin (0.820 ± 0.180), and 1 μM PFHpA + 1 μM PFPA + 10 – 50 μM cisplatin (10 μM: 0.833 ± 0.130, 25 μM: 0.787 ± 0.132, 50 μM: 0.825 ± 0159). Data shown are normalized to the vehicle control; n= at least 4 independent experiments in duplicate. Significant differences between PFAS mixture + chemotherapy treatment group versus vehicle group at each respective chemotherapy dose are denoted by * (*p* < 0.05).

**
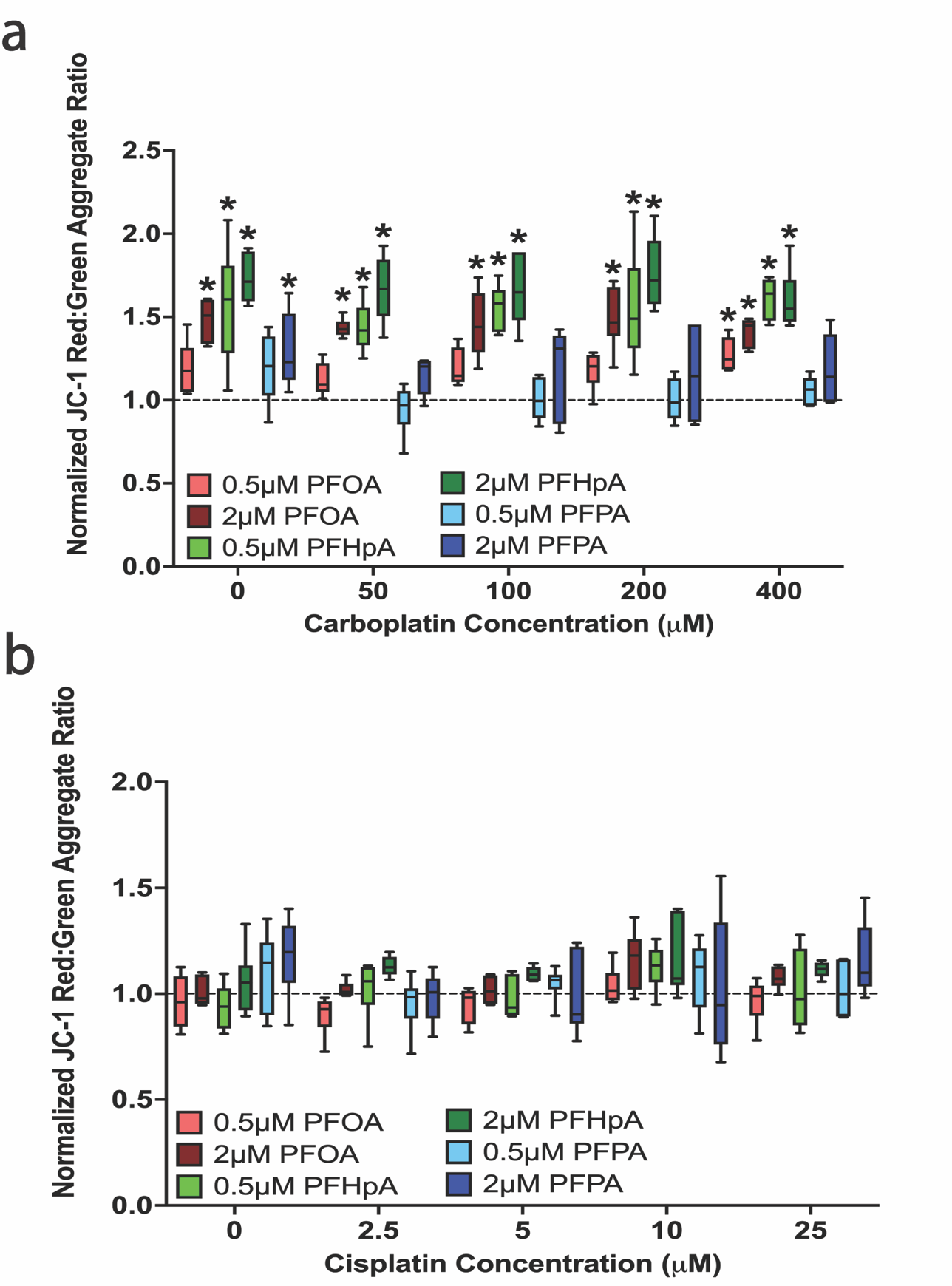
**

**Figure S11**: ΔΨ_m_ increased in HEC-1 cells exposed to PFAS then treated with carboplatin, but not cisplatin. (a) In HEC-1 cells exposed to PFAS + carboplatin, ΔΨ_m_ increased compared to the vehicle control (dashed line) in the following groups: 0.5 μM PFOA + 400 μM carboplatin (1.274 ± 0.101), 2 μM PFOA + 0 – 400 μM carboplatin (0 μM: 1.482 ± 0.124, 50 μM: 1.434 ± 0.054, 100 μM: 1.456 ± 0.197, 200 μM: 1.493 ± 0.185, 400 μM: 1.410 ± 0.087), 0.5 μM PFHpA + 0 – 400 μM carboplatin (0 μM: 1.571 ± 0.347, 50 μM: 1.439 ± 0.149, 100 μM: 1.558 ± 0.137, 200 μM: 1.552 ± 0.338, 400 μM: 1.612 ± 0.126), and 2 μM PFHpA + 0 – 400 μM carboplatin (0 μM: 1.732 ± 0.155, 50 μM: 1.667 ± 0.196, 100 μM: 1.658 ± 0.215, 200 μM: 1.763 ± 0.212, 400 μM: 1.601 ± 0.179). (b) ΔΨ_m_ was unchanged in HEC-1 cells exposed to PFAS then treated with cisplatin. Data shown are mean ± SD; n=3 independent experiments in duplicate. Significant differences between PFAS + chemotherapy treatment group versus vehicle group at each respective chemotherapy dose are denoted by * (*p* < 0.05).

**
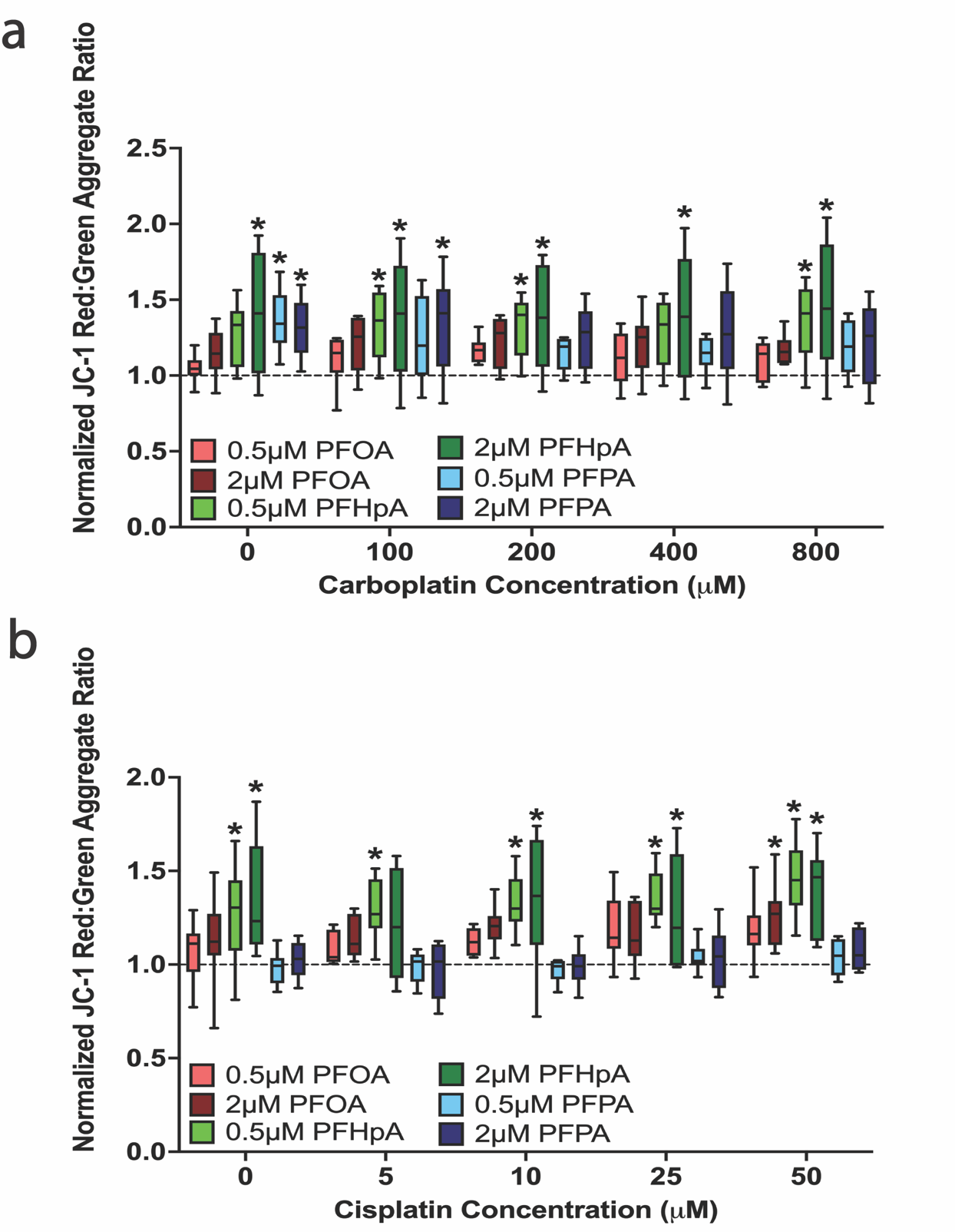
**

**Figure S12**: ΔΨ_m_ increased in Ishikawa cells exposed to PFAS then treated with platinum-based chemotherapy. (a) In Ishikawa cells exposed to PFAS + carboplatin, ΔΨ_m_ increased compared to the vehicle control (dashed line) in the following groups: 0.5 μM PFHpA + 100, 200, and 800 μM carboplatin (100 μM: 1.336 ± 0.223, 200 μM: 1.332 ± 0.201, 800 μM: 1.358 ± 0.247), 2 μM PFHpA + 0 – 800 μM carboplatin (0 μM: 1.413 ± 0.394, 100 μM: 1.378 ± 0.385, 200 μM: 1.387 ± 0.333, 400 μM: 1.393 ± 0.406, 800 μM: 1.461 ± 0.411), 0.5 μM PFPA + 0 μM carboplatin (1.354 ± 0.199), 2 μM PFPA + 0 μM carboplatin (1.305 ± 0.192). (b) In Ishikawa cells exposed to PFAS + cisplatin, ΔΨ_m_ increased compared to the vehicle control (dashed line) in the following groups: 2 μM PFOA + 50 μM cisplatin (50 μM: 1.258 ± 0.171), 0.5 μM PFHpA + 0 – 50 μM cisplatin (0 μM: 1.268 ± 0.265, 5 μM: 1.298 ± 0.164, 10 μM: 1.322 ± 0.152, 25 μM: 1.359 ± 0.139, 50 μM: 1.463 ± 0.196), and 2 μM PFHpA + 0, 10, 25, and 50 μM cisplatin (0 μM: 1.352 ± 0.301, 10 μM: 1.336 ± 0.357, 25 μM: 1.272 ± 0.302, 50 μM: 1.385 ± 0.229). Data shown are mean ± SD; n=4 independent experiments in duplicate. Significant differences between PFAS + chemotherapy treatment group versus vehicle group at each respective chemotherapy dose are denoted by * (*p* < 0.05).
